# Supplementary material for: Opioid-sparing anesthesia versus opioid-free anesthesia for postoperative recovery quality in breast cancer surgery patients: A systematic review and Bayesian network meta-analysis
Source: PLoS One. 2025 Oct 24;20(10):e0334614. doi: 10.1371/journal.pone.0334614 (PMC12551851; doi:10.1371/journal.pone.0334614)
Supplement: S1 Text — Search terms and database strategies. (DOCX) [file pone.0334614.s002.docx]

**Search Strategy**

**Pubmed:**

1. ("analgesics, opioid"[MeSH] OR opioid*[TIAB] OR opiate*[TIAB] OR fentanyl[TIAB] OR morphine[TIAB] OR oxycodone[TIAB])

2. (free[TIAB] OR spar*[TIAB] OR reduc*[TIAB] OR minimi*[TIAB] OR non-opioid*[TIAB] OR "low dose"[TIAB] OR "low-dose"[TIAB])

3. #1 AND #2

4. ("opioid free"[TIAB] OR "opioid spar*"[TIAB] OR "non-opioid"[TIAB] OR "low-dose opioid"[TIAB] OR "low opioid"[TIAB] OR "reduced opioid"[TIAB])

5. #3 OR #4

6. ("anesthesia"[MeSH] OR "anesthetics"[MeSH] OR "analgesia"[MeSH] OR "nerve block"[MeSH] OR anesthes*[TIAB] OR anaesthes*[TIAB] OR analges*[TIAB] OR "multimodal analgesia"[TIAB])

7. ("dexmedetomidine"[TIAB] OR "ketamine"[TIAB] OR "lidocaine"[TIAB] OR "NSAIDs"[TIAB] OR "acetaminophen"[TIAB])

8. #6 OR #7

9. ("Mastectomy"[MeSH] OR "Breast Neoplasms/surgery"[MeSH] OR "Mammaplasty"[MeSH] OR "breast surg*"[TIAB] OR mastectom*[TIAB] OR lumpectomy[TIAB] OR "breast reconstruction"[TIAB] OR "breast cancer surgery"[TIAB])

10. ("quality of recovery"[TIAB] OR "QoR-40"[TIAB] OR "QoR-15"[TIAB] OR "recovery score"[TIAB] OR "pain, postoperative"[MeSH])

11. #5 AND #8 AND #9 AND #10

12. ("randomized controlled trial"[PT] OR "controlled clinical trial"[PT] OR randomized[TIAB] OR randomised[TIAB] OR trial[TIAB] OR groups[TIAB])

13. #11 AND #12)

[**Cochrane**](https://www.cochranelibrary.com/library)**:**

(( (free OR spar* OR reduc* OR minimi* OR non-opioid* OR low dose OR low-dose))

OR (opioid free OR opioid spar* OR non-opioid OR low-dose opioid OR low opioid OR reduced opioid))

AND

((anesthesia OR anesthetics OR analgesia OR nerve block OR anesthes* OR anaesthes* OR analges* OR multimodal analgesia)

OR (dexmedetomidine OR ketamine OR lidocaine OR NSAIDs OR acetaminophen OR magnesium sulfate))

AND

(mastectomy OR "breast neoplasms surgery" OR mammaplasty OR breast surgery* OR mastectomy* OR lumpectomy OR breast reconstruction OR breast cancer surgery)

AND

(quality of recovery OR QoR-40 OR QoR-15 OR recovery score OR "pain postoperative")

AND

(randomized controlled trial OR controlled clinical trial OR randomized OR randomised OR trial OR groups)

**Embase:**

1. 'opioid analgesic agent'/exp OR opioid*:ti,ab OR opiate*:ti,ab OR fentanyl:ti,ab OR morphine:ti,ab OR oxycodone:ti,ab

2. free:ti,ab OR spar*:ti,ab OR reduc*:ti,ab OR minimi*:ti,ab OR 'non-opioid*':ti,ab OR 'low dose':ti,ab OR 'low-dose':ti,ab

3. #1 AND #2

4. 'opioid free':ti,ab OR 'opioid spar*':ti,ab OR 'non-opioid':ti,ab OR 'low-dose opioid':ti,ab OR 'low opioid':ti,ab OR 'reduced opioid':ti,ab

5. #3 OR #4

6. 'anesthesia'/exp OR 'anesthetic agent'/exp OR 'analgesia'/exp OR 'nerve block'/exp OR anesthes*:ti,ab OR anaesthes*:ti,ab OR analges*:ti,ab OR 'multimodal analgesia':ti,ab

7. dexmedetomidine:ti,ab OR ketamine:ti,ab OR lidocaine:ti,ab OR 'nsaid'/exp OR 'acetaminophen'/exp

8. #6 OR #7

9. 'mastectomy'/exp OR 'breast cancer'/exp AND [surgery]/lim OR 'mammaplasty'/exp OR 'breast surgery':ti,ab OR mastectom*:ti,ab OR lumpectomy:ti,ab OR 'breast reconstruction':ti,ab OR 'breast cancer surgery':ti,ab

10. 'quality of recovery':ti,ab OR 'qor-40':ti,ab OR 'qor-15':ti,ab OR 'recovery score':ti,ab OR 'postoperative pain'/exp

11. #5 AND #8 AND #9 AND #10

12. 'randomized controlled trial'/de OR 'controlled clinical trial'/de OR randomized:ti,ab OR randomised:ti,ab OR trial:ti,ab OR groups:ti,ab

13. #11 AND #12

**Web of science:**

#1 TS=("opioid analgesics" OR opioid* OR opiate* OR fentanyl OR morphine OR oxycodone)

#2 TS=(free OR spar* OR reduc* OR minimi* OR "non-opioid*" OR "low dose" OR "low-dose")

#3 #1 AND #2

#4 TS=("opioid free" OR "opioid spar*" OR "non-opioid" OR "low-dose opioid" OR "low opioid" OR "reduced opioid")

#5 #3 OR #4

#6 TS=("anesthesia" OR "anesthetics" OR "analgesia" OR "nerve block" OR anesthes* OR anaesthes* OR analges* OR "multimodal analgesia")

#7 TS=(dexmedetomidine OR ketamine OR lidocaine OR "NSAIDs" OR acetaminophen)

#8 #6 OR #7

#9 TS=("mastectomy" OR "breast cancer surgery" OR "mammaplasty" OR "breast surg*" OR mastectom* OR lumpectomy OR "breast reconstruction")

#10 TS=("quality of recovery" OR "QoR-40" OR "QoR-15" OR "recovery score" OR "postoperative pain")

#11 #5 AND #8 AND #9 AND #10

#12 TS=("randomized controlled trial" OR "controlled clinical trial" OR randomized OR randomised OR trial OR groups)

#13 #11 AND #12
