# Supplementary material for: Opioid-sparing anesthesia versus opioid-free anesthesia for postoperative recovery quality in breast cancer surgery patients: A systematic review and Bayesian network meta-analysis
Source: PLoS One. 2025 Oct 24;20(10):e0334614. doi: 10.1371/journal.pone.0334614 (PMC12551851; doi:10.1371/journal.pone.0334614)
Supplement: S2 Text — The reasons for exclusion after full-text review. (DOCX) [file pone.0334614.s003.docx]

**The reasons for exclusion after full-text review**

**Non-conformity with grouping criteria:**

1. Kendall, MC, McCarthy, RJ, Panaro, S, Goodwin, E, Bialek, JM, Nader, A, De Oliveira, GS. The Effect of Intraoperative Systemic Lidocaine on Postoperative Persistent Pain Using Initiative on Methods, Measurement, and Pain Assessment in Clinical Trials Criteria Assessment Following Breast Cancer Surgery: A Randomized, Double-Blind, Placebo-Controlled Trial. PAIN PRACT. 2018-03-01; 18 (3): 350-359. doi: 10.1111/papr.12611. PMID: 28691269;
2. Kulturoglu, G, Altinsoy, S, Ergil, J, Ozkan, D, Ozguner, Y. Investigation of the analgesic effects of rhomboid intercostal and pectoral nerve blocks in breast surgery. J ANESTH. 2024-10-01; 38 (5): 584-590. doi: 10.1007/s00540-024-03351-3. PMID: 38777932;
3. Yu, L, Zhou, Q, Li, W, Zhang, Q, Cui, X, Chang, Y, Wang, Q. Effects of Esketamine Combined with Ultrasound-Guided Pectoral Nerve Block Type II on the Quality of Early Postoperative Recovery in Patients Undergoing a Modified Radical Mastectomy for Breast Cancer: A Randomized Controlled Trial. J Pain Res. 2022-01-01; 15 3157-3169. doi: 10.2147/JPR.S380354. PMID: 36311293;
4. Zhao, Y, Jin, W, Pan, P, Feng, S, Fu, D, Yao, J. Ultrasound-guided transversus thoracic muscle plane-pectoral nerve block for postoperative analgesia after modified radical mastectomy: a comparison with the thoracic paravertebral nerve block. Perioper Med (Lond). 2022-07-27; 11 (1): 39. doi: 10.1186/s13741-022-00270-3. PMID: 35883207;
5. Doan, LV, Li, A, Brake, L, et al. Single-Dose of Postoperative Ketamine for Postoperative Pain After Mastectomy: A Pilot Randomized Controlled Trial. J Pain Res. 2023; 16 881-892. doi: 10.2147/JPR.S389564
6. Dinh, KH, McAuliffe, PF, Boisen, M, Esper, SA, Subramaniam, K, Steiman, JG, Soran, A, Johnson, RR, Holder-Murray, JM, Diego, EJ. Post-operative Nausea and Analgesia Following Total Mastectomy is Improved After Implementation of an Enhanced Recovery Protocol. ANN SURG ONCOL. 2020-11-01; 27 (12): 4828-4834. doi: 10.1245/s10434-020-08880-1. PMID: 32748151;
7. Do, U, El-Kefraoui, C, Pook, M, Balvardi, S, Barone, N, Nguyen-Powanda, P, Lee, L, Baldini, G, Feldman, LS, Fiore, JF, Alhashemi, M, Antoun, A, Barkun, JS, Brecht, KM, Chaudhury, PK, Deckelbaum, D, Di Lena, E, Dumitra, S, Elhaj, H, Fata, P, Fleiszer, D, Fried, GM, Grushka, J, Kaneva, P, Khwaja, K, Lapointe-Gagner, M, McKendy, KM, Meguerditchian, AN, Meterissian, SH, Montgomery, H, Rajabiyazdi, F, Safa, N, Touma, N, Tremblay, F. Feasibility of Prospectively Comparing Opioid Analgesia With Opioid-Free Analgesia After Outpatient General Surgery: A Pilot Randomized Clinical Trial. JAMA Netw Open. 2022-07-01; 5 (7): e2221430. doi: 10.1001/jamanetworkopen.2022.21430. PMID: 35849399;
8. Wang, X, Ran, G, Chen, X, Xie, C, Wang, J, Liu, X, Lu, Y, Fang, W. The Effect of Ultrasound-Guided Erector Spinae Plane Block Combined with Dexmedetomidine on Postoperative Analgesia in Patients Undergoing Modified Radical Mastectomy: A Randomized Controlled Trial. PAIN THER. 2021-06-01; 10 (1): 475-484. doi: 10.1007/s40122-020-00234-9. PMID: 33475952;
9. Datchinamourthy, T, Bhoi, D, Chhabra, A, Mohan, VK, Kumar, KR, Ranganathan, P. Comparative evaluation of continuous infusion versus programmed intermittent bolus techniques in erector spinae plane block in modified radical mastectomy - A preliminary randomised controlled trial. INDIAN J ANAESTH. 2024-03-01; 68 (3): 273-279. doi: 10.4103/ija.ija_922_23. PMID: 38476552;
10. Jiang, CW, Liu, F, Zhou, Q, Deng, W. Comparison of rhomboid intercostal nerve block, erector spinae plane block and serratus plane block on analgesia for modified radical mastectomy: A prospective randomised controlled trial. INT J CLIN PRACT. 2021-10-01; 75 (10): e14539. doi: 10.1111/ijcp.14539. PMID: 34133831;
11. Ilfeld, BM, Said, ET, Gabriel, RA, Curran, BP, Swisher, MW, Jacobsen, GR, Wallace, AM, Doucet, J, Adams, LM, Ventro, GJ, Abdullah, B, Finneran, JJ. Wearable, noninvasive, pulsed shortwave (radiofrequency) therapy for analgesia and opioid sparing following outpatient surgery: A proof-of-concept case series. PAIN PRACT. 2023-06-01; 23 (5): 553-558. doi: 10.1111/papr.13188. PMID: 36463434;
12. Do, U, Pook, M, Najafi, T, Rajabiyazdi, F, El-Kefraoui, C, Balvardi, S, Barone, N, Elhaj, H, Nguyen-Powanda, P, Lee, L, Baldini, G, Feldman, LS, Fiore, JF. S110-Opioid-free analgesia after outpatient general surgery: A qualitative study focused on the perspectives of patients and clinicians involved in a pilot trial. SURG ENDOSC. 2023-03-01; 37 (3): 2269-2280. doi: 10.1007/s00464-022-09472-8. PMID: 35918552;
13. Sethi, D, Ramakrishnan, P, Khurana, GK, Arora, A. Outcome of perioperative 24-hour infusion of intravenous lignocaine on pain and QoR-15 scores after breast cancer surgery-A randomised controlled trial. INDIAN J ANAESTH. 2023-02-01; 67 (Suppl 2): S113-S119. doi: 10.4103/ija.ija_126_22. PMID: 37122935;
14. Wu, Y, Kang, Y, Li, Y, Fu, B. Impact of Ultrasound-Guided Deep Serratus Anterior Plane Block Combined With Dexmedetomidine as an Adjuvant to Ropivacaine Inpatient Quality of Recovery Scores Undergoing Modified Radical Mastectomy: A Randomized Controlled Trial. Front Oncol. 2022-01-01; 12 858030. doi: 10.3389/fonc.2022.858030. PMID: 35433468;
15. Zhao, Z, Xu, Q, Chen, Y, Liu, C, Zhang, F, Han, Y, Cao, J. The effect of low-dose ketamine on postoperative quality of recovery in patients undergoing breast cancer surgery: A randomised, placebo-controlled trial. INT J CLIN PRACT. 2021-12-01; 75 (12): e15010. doi: 10.1111/ijcp.15010. PMID: 34807494;
16. Hetta, DF, Elgalaly, NA, Hetta, HF, Fattah Mohammad, MA. Preoperative Duloxetine to improve acute pain and quality of recovery in patients undergoing modified radical mastectomy: A dose-ranging randomized controlled trial. J CLIN ANESTH. 2020-12-01; 67 110007. doi: 10.1016/j.jclinane.2020.110007. PMID: 32847776;
17. Rokhtabnak, F, Sayad, S, Izadi, M, Djalali Motlagh, S, Rahimzadeh, P. Pain Control After Mastectomy in Transgender Patients: Ultrasound-guided Pectoral Nerve Block II Versus Conventional Intercostal Nerve Block: A Randomized Clinical Trial. Anesth Pain Med. 2021-10-01; 11 (5): e119440. doi: 10.5812/aapm.119440. PMID: 35070905;
18. Hartford, LB, Van Koughnett, JAM, Murphy, PB, Knowles, SA, Wigen, RB, Allen, LJ, Clarke, CFM, Brackstone, M, Gray, DK, Maciver, AH. The Standardization of Outpatient Procedure (STOP) Narcotics: A Prospective Health Systems Intervention to Reduce Opioid Use in Ambulatory Breast Surgery. ANN SURG ONCOL. 2019-10-01; 26 (10): 3295-3304. doi: 10.1245/s10434-019-07539-w. PMID: 31342371;
19. Wang, M, Niu, SJ, Wu, J, Zhong, YW, Lu, ZY, Fu, Q, Li, BB. Impact of Posterior Quadratus Lumborum Block on Acute Pain Relief and Chronic Pain Prevention in Breast Cancer Surgery. Pain Ther. 2025-05-29; doi: 10.1007/s40122-025-00740-8. PMID: 40439825;
20. Kamiya, Y, Hasegawa, M, Yoshida, T, Takamatsu, M, Koyama, Y. Impact of pectoral nerve block on postoperative pain and quality of recovery in patients undergoing breast cancer surgery: A randomised controlled trial. EUR J ANAESTH. 2018-03-01; 35 (3): 215-223. doi: 10.1097/EJA.0000000000000762. PMID: 29227351;
21. Abu Elyazed, MM, Mostafa, SF. Continuous Pectoral Nerve Block Compared With Continuous Thoracic Paravertebral Block and Intravenous Opioid Analgesia for the Postoperative Analgesic Efficacy in Patients Undergoing Modified Radical Mastectomy: A Prospective Randomized Trial. CLIN J PAIN. 2021-05-01; 37 (5): 359-365. doi: 10.1097/AJP.0000000000000932. PMID: 33734144;
22. Kumar, M, Gupta, R, Dinkar, PK, Abbas, H. A Comparative Study of Morphine and Clonidine as an Adjunct to Ropivacaine in Paravertebral Block for Modified Radical Mastectomy. Cureus. 2023-08-01; 15 (8): e42950. doi: 10.7759/cureus.42950. PMID: 37667700;
23. Ahuja, D, Kumar, V, Gupta, N, Bharati, SJ, Garg, R, Mishra, S, Khan, MA, Bhatnagar, S. Comparison of the Efficacy of UltrasoundGuided Serratus Anterior Plane Block Versus Erector Spinae Plane Block for Postoperative Analgesia After Modified Radical Mastectomy: A Randomised Controlled Trial. Turk J Anaesthesiol Reanim. 2022-12-01; 50 (6): 435-442. doi: 10.5152/TJAR.2022.21127. PMID: 36511493;

**No QoR results:**

1. Grasso, A, Orsaria, P, Costa, F, D'Avino, V, Caredda, E, Hazboun, A, Carino, R, Pascarella, G, Altomare, M, Buonomo, OC, Agrò, FE, Altomare, V. Ultrasound-guided Interfascial Plane Blocks for Non-anesthesiologists in Breast Cancer Surgery: Functional Outcomes and Benefits. ANTICANCER RES. 2020-04-01; 40 (4): 2231-2238. doi: 10.21873/anticanres.14185. PMID: 32234919;
2. Gabriel, RA, Curran, BP, Swisher, MW, Sztain, JF, Tsuda, PS, Said, ET, Alexander, B, Finneran, JJ, Abramson, WB, Black, JR, Wallace, AM, Blair, S, Donohue, MC, Abdullah, B, Xu, NY, Cha, BJ, Ilfeld, BM. Paravertebral versus Pectoralis-II (Interpectoral and Pectoserratus) Nerve Blocks for Postoperative Analgesia after Nonmastectomy Breast Surgery: A Randomized, Controlled, Observer-masked Noninferiority Trial. ANESTHESIOLOGY. 2024-12-01; 141 (6): 1039-1050. doi: 10.1097/ALN.0000000000005207. PMID: 39186671;
3. Sulak, M, Ahiskalioglu, A, Yayik, A, Karadeniz, E, Celik, M, Demir, U, Ari, M, Alici, H. The effect of ultrasound-guided serratus plane block on the quality of life in patients undergoing modified radical mastectomy and axillary lymph node dissection: a randomized controlled study. ANAESTH INTENSIVE TH. 2022-01-01; 54 (1): 48-55. doi: 10.5114/ait.2022.114203. PMID: 35266378;
4. Wittayapairoj, A, Wittayapairoj, K, Vechvitvarakul, M. Effect of bilateral ultrasound-guided erector spinae plane block on postoperative pain after open lumbar spinal surgery: a double-blind, randomized controlled trial. EUR SPINE J. 2023-02-01; 32 (2): 420-427. doi: 10.1007/s00586-022-07494-3. PMID: 36515773;
5. Nikolić, A, Stošić, M, Živadinović, J, Gmijović, M, ĐorĐević, M, Janković, R, Karanikolić, A, Stošić, B. The impact of ultrasound-guided erector spinae plane block on hemodynamic stability and postoperative pain in patients undergoing modified radical mastectomy for breast cancer. EUR REV MED PHARMACO. 2024-04-01; 28 (8): 3120-3134. doi: 10.26355/eurrev_202404_36028. PMID: 38708471;
6. Sivrikoz, N, Turhan, Ö, Ali, A, Altun, D, Tükenmez, M, Sungur, Z. Paravertebral block versus erector spinae plane block for analgesia in modified radical mastectomy: a randomized, prospective, double-blind study. MINERVA ANESTESIOL. 2022-12-01; 88 (12): 1003-1012. doi: 10.23736/S0375-9393.22.16625-3. PMID: 36282220;
7. Kim, DH, Kim, S, Kim, CS, Lee, S, Lee, IG, Kim, HJ, Lee, JH, Jeong, SM, Choi, KT. Efficacy of Pectoral Nerve Block Type II for Breast-Conserving Surgery and Sentinel Lymph Node Biopsy: A Prospective Randomized Controlled Study. PAIN RES MANAG. 2018-01-01; 2018 4315931. doi: 10.1155/2018/4315931. PMID: 29861803;
8. Goswami, S, Kundra, P, Bhattacharyya, J. Pectoral nerve block1 versus modified pectoral nerve block2 for postoperative pain relief in patients undergoing modified radical mastectomy: a randomized clinical trial. BRIT J ANAESTH. 2017-10-01; 119 (4): 830-835. doi: 10.1093/bja/aex201. PMID: 29121291;
9. Romagnoli, F, Trotta, V, Stancampiano, P, Colalongo, C, Grugni, L, Bortolin, G, Zonta, S. Feasibility and efficacy of routinary PECs 1 block on perioperative and postoperative pain control in breast surgery: a prospective non-controlled trial in a medium-volume breast unit. UPDATES SURG. 2023-08-01; 75 (5): 1297-1303. doi: 10.1007/s13304-023-01517-4. PMID: 37095357;
10. Senapathi, TGA, Widnyana, IMG, Aribawa, IGNM, Jaya, AAGPS, Junaedi, IMD. Combined ultrasound-guided Pecs II block and general anesthesia are effective for reducing pain from modified radical mastectomy. J Pain Res. 2019-01-01; 12 1353-1358. doi: 10.2147/JPR.S197669. PMID: 31114311;
11. Kasimahanti, R, Arora, S, Bhatia, N, Singh, G. Ultrasound-guided single- vs double-level thoracic paravertebral block for postoperative analgesia in total mastectomy with axillary clearance. J CLIN ANESTH. 2016-09-01; 33 414-21. doi: 10.1016/j.jclinane.2016.01.027. PMID: 27555203;
12. Gürkan, Y, Aksu, C, Kuş, A, Yörükoğlu, UH. Erector spinae plane block and thoracic paravertebral block for breast surgery compared to IV-morphine: A randomized controlled trial. J CLIN ANESTH. 2020-02-01; 59 84-88. doi: 10.1016/j.jclinane.2019.06.036. PMID: 31280100;
13. El-Megeed, M, Hassan, A, Ashour, T, Youssef, A. Comparative Study between the Effect of Ultrasound Guided Pectoral Nerve Block (PEC 1) versus Serratus Anterior Plane Block (SAPB) For Postoperative Analgesia in Modified Radical Mastectomy QJM-INT J MED. 2021-10-01; 114 (Supple1): doi: 10.1093/qjmed/hcab086.061.
14. Hassan, ME, Mahran, E. Effect of magnesium sulfate with ketamine infusions on intraoperative and postoperative analgesia in cancer breast surgeries: a randomized double-blind trial. Braz J Anesthesiol. 2023-01-01; 73 (2): 165-170. doi: 10.1016/j.bjane.2021.07.015. PMID: 34332956;
15. Aboalsoud, R, Arida, E, Sabry, L, Elmolla, A, Mohammad Ghoneim, H. The effect of opioid free versus opioid based anaesthesia on breast cancer pain score and immune response Egypt J Anaesth. 2021-01-01; 37 (1): 472-482. doi: 10.1080/11101849.2021.1983366.
16. Kumar, P, Singh, A, Sharma, J, Parshad, S, Johar, S, Kaur, K. Assessment of ultrasound guided erector spinae plane block for early post-operative analgesia for modified radical mastectomy: a prospective, randomized, controlled study. Med Gas Res. 2024-12-01; 14 (4): 201-205. doi: 10.4103/mgr.mgr_74_20. PMID: 39073328;
17. Cylwik, J, Celińska-Spodar, M, Buda, N. Evaluation of the Efficacy of Pectoral Nerve-2 Block (PECS 2) in Breast Cancer Surgery. J Pers Med. 2023-09-24; 13 (10): doi: 10.3390/jpm13101430. PMID: 37888041;
18. Elewa, AM, Faisal, M, Sjöberg, F, Abuelnaga, ME. Comparison between erector spinae plane block and paravertebral block regarding postoperative analgesic consumption following breast surgery: a randomized controlled study. BMC Anesthesiol. 2022-06-18; 22 (1): 189. doi: 10.1186/s12871-022-01724-3. PMID: 35717148;
19. Wei, Z, Lei, GY, Wu, LL, Xi, CH, Yin, Y, Wang, GY. [Effect of ultrasound-guided serratus plane block combined with pectoral nerve block I on postoperative analgesia after radical mastectomy]. Zhonghua Yi Xue Za Zhi. 2022-08-09; 102 (29): 2278-2282. doi: 10.3760/cma.j.cn112137-20220513-01048. PMID: 35927059;
20. Qian, XL, Li, P, Chen, YJ, et al. Opioid Free Total Intravenous Anesthesia With Dexmedetomidine-Esketamine-Lidocaine for Patients Undergoing Lumpectomy. J Clin Med Res. 2023; 15 (8-9): 415-422. doi: 10.14740/jocmr5000
21. Liu, PC, Su, FW, Tsai, YF, Lin, YS, Sung, CS, Tseng, LM, Teng, WN. Multimodal analgesia with thoracic paravertebral block decrease pain and side effects in mastectomy patients. J Chin Med Assoc. 2025-06-01; 88 (6): 486-491. doi: 10.1097/JCMA.0000000000001218. PMID: 39934978;
22. Cho, A. Effects of Intraoperative Low-Dose Ketamine on Persistent Postsurgical Pain after Breast Cancer Surgery: A Prospective, Randomized, Controlled, Double-Blind Study PAIN PHYSICIAN. 2020-01-14; 1;23 (1;1): 37-47. doi: 10.36076/ppj.2020/23/37.
23. Hu, H, Luo, Z, Li, B, Wang, T, Wu, T, Li, B, Song, X. Effect of ultrasound-guided PecS II block on the incidence of chronic postmastectomy pain in patients after radical mastectomy: A randomized controlled trial. Saudi J Anaesth. 2025-01-01; 19 (2): 235-242. doi: 10.4103/sja.sja_398_24. PMID: 40255352;
24. Su, YH, Luo, DC, Pang, Y. Effects of intraoperative Magnesium sulfate infusion on emergency agitation during general anesthesia in patients undergoing radical mastectomy: a randomized controlled study. BMC Anesthesiol. 2023-09-26; 23 (1): 326. doi: 10.1186/s12871-023-02288-6. PMID: 37749511;
25. Cvetković, A, Ivan, M, Milan, Ž, Jevrić, M, Bukumiric, Z, Dijana, M, Jokić, A, Damjana, B, Buta, M. Comparing postoperative pain control after modified radical mastectomy: a pilot study of ultra-sound guided erector spinae plane block vs intraoperative tramadol administration in oncology patients. J Cancer Res Clin Oncol. 2025-04-15; 151 (4): 140. doi: 10.1007/s00432-025-06197-8. PMID: 40232315;
26. Devrajan, G, Chhabra, PH, Guria, S, Gupta, K. Comparison of intravenous with perineural dexamethasone for ultrasound-guided erector spinae plane block in patients undergoing modified radical mastectomy-A randomized control trial. J Anaesthesiol Clin Pharmacol. 2025-01-01; 41 (2): 357-362. doi: 10.4103/joacp.joacp_134_24. PMID: 40248794;
27. Lin, X, Cai, Y, Chen, X, Lin, J, He, Y, Xie, L, Jiang, X, Chen, Y. Analgesia and stress attenuation of ultrasound-guided modified pectoral nerve block type-II with different volumes of 0.3% ropivacaine in patients undergoing modified radical mastectomy for breast cancer: A prospective parallel randomized double-blind controlled clinical trial. J CLIN PHARM THER. 2022-10-01; 47 (10): 1676-1683. doi: 10.1111/jcpt.13720. PMID: 35765728;
28. Chai, B, Yu, H, Qian, Y, et al. Comparison of Postoperative Pain in 70 Women with Breast Cancer Following General Anesthesia for Mastectomy with and without Serratus Anterior Plane Nerve Block. Med Sci Monit. 2022; 28 e934064. doi: 10.12659/MSM.934064
29. Gassman, AA, Yoon, AP, Maxhimer, JB, Sanchez, I, Sethi, H, Cheng, KW, Tseng, CY, Festekjian, JH, Da Lio, AL, Crisera, CA. Comparison of postoperative pain control in autologous abdominal free flap versus implant-based breast reconstructions. PLAST RECONSTR SURG. 2015-02-01; 135 (2): 356-367. doi: 10.1097/PRS.0000000000000989. PMID: 25626783;
30. Kim, WJ, Lim, W. Efficacy of erector spinae plane block with opioid-sparing analgesic technique in breast-conserving surgery. ANN SURG TREAT RES. 2021-05-01; 100 (5): 253-259. doi: 10.4174/astr.2021.100.5.253. PMID: 34012942;
31. Mazzinari, G, Rovira, L, Casasempere, A, Ortega, J, Cort, L, Esparza-Miñana, JM, Belaouchi, M. Interfascial block at the serratus muscle plane versus conventional analgesia in breast surgery: a randomized controlled trial. REGION ANESTH PAIN M. 2019-01-01; 44 (1): 52-58. doi: 10.1136/rapm-2018-000004. PMID: 30640653;
32. Tavares Mendonça, F, de Assis Feitosa Junior, A, Nogueira, H, Roncolato, H, Sousa Goveia, C. Efficacy of type-I and type-II pectoral nerve blocks (PECS I and II) in patients undergoing mastectomy: a prospective randomised clinical trial. ANAESTH INTENSIVE TH. 2022-01-01; 54 (4): 302-309. doi: 10.5114/ait.2022.121096. PMID: 36458667;
33. Kurien, RK, Salins, SR, Jacob, PM, Thomas, K. Utility of Pecs Block for Perioperative Opioid-Sparing Analgesia in Cancer-Related Breast Surgery: A Randomized Controlled Trial. INDIA J SURG ONCOL. 2021-12-01; 12 (4): 713-721. doi: 10.1007/s13193-021-01382-w. PMID: 35110894;
34. Leite, ALDS, Rocha, FTR, Oliveira, MJC, Barros, AV, Santos, SMLD, Silva, AMRD, Silvestre, DWA, Folha Filho, EAC, Ferro, CC, Bezerra, TS, Fachin, LP, Santos, DC, Fraga, CAC, Sales-Marques, C. Impact of Pectoralis Nerve Block (PECS) on postoperative pain in patients submitted to mastectomy with lymphadenectomy. Rev Col Bras Cir. 2022-01-01; 49 e20223366. doi: 10.1590/0100-6991e-20223366-en. PMID: 36515333;
35. Lin, ZM, Li, MH, Zhang, F, Li, X, Shao, CL, Li, XY, Wang, DX. Thoracic Paravertebral Blockade Reduces Chronic Postsurgical Pain in Breast Cancer Patients: A Randomized Controlled Trial. PAIN MED. 2020-12-25; 21 (12): 3539-3547. doi: 10.1093/pm/pnaa270. PMID: 33111950;
36. M, N, Pandey, RK, Sharma, A, Darlong, V, Punj, J, Sinha, R, Singh, PM, Hamshi, N, Garg, R, Chandralekha, C, Srivastava, A. Pectoral nerve blocks to improve analgesia after breast cancer surgery: A prospective, randomized and controlled trial. J CLIN ANESTH. 2018-03-01; 45 12-17. doi: 10.1016/j.jclinane.2017.11.027. PMID: 29241077;

**Non RCT:**

1. Haddock, NT, Cummins, S, Lakatta, AC, Teotia, SS, Farr, D. Enhanced Recovery After Surgery (ERAS) With Exparel in Tissue Expander-based Breast Reconstruction Following Mastectomy. AESTHET SURG J. 2024-08-16; 44 (Supple1): S15-S21. doi: 10.1093/asj/sjae003. PMID: 39147381;
2. Haddock, NT, Garza, R, Boyle, CE, Liu, Y, Teotia, SS. Defining Enhanced Recovery Pathway with or without Liposomal Bupivacaine in DIEP Flap Breast Reconstruction. PLAST RECONSTR SURG. 2021-11-01; 148 (5): 948-957. doi: 10.1097/PRS.0000000000008409. PMID: 34705768;
3. Lee, JE, Park, YJ, Lee, JW. Ropivacaine continuous wound infusion after mastectomy with immediate autologous breast reconstruction: A retrospective observational study. MEDICINE. 2021-06-18; 100 (24): e26337. doi: 10.1097/MD.0000000000026337. PMID: 34128878;
4. Di Benedetto, P, Pelli, M, Loffredo, C, La Regina, R, Policastro, F, Fiorelli, S, De Blasi, RA, Coluzzi, F, Rocco, M. Opioid-free anesthesia versus opioid-inclusive anesthesia for breast cancer surgery: a retrospective study. 2021-10-09; 1 (1): 6. doi: 10.1186/s44158-021-00008-5. PMID: 37386556;
5. Assaf, GR, Yared, F, Dib, MJ, Mouawad, T, Tarabay, O, Noujeim, JP, El-Helou, E, Kaady, J, Abboud, B. Efficacy of opioid-free anesthesia in modified radical mastectomy: a cross-sectional observational study. Ann Med Surg (Lond). 2023-09-01; 85 (9): 4289-4292. doi: 10.1097/MS9.0000000000000718. PMID: 37663699;
6. Fanelli, A, Torrano, V, Cozowicz, C, Mariano, ER, Balzani, E. The opioid sparing effect of erector spinae plane block for various surgeries: a meta-analysis of randomized-controlled trials. MINERVA ANESTESIOL. 2021-08-01; 87 (8): 903-914. doi: 10.23736/S0375-9393.21.15356-8. PMID: 33982985;
7. Quan, Y, Liu, Y, Li, G, Liu, Z. Clinical application of ultrasound-guided thoracic nerve block in the operation of benign breast tumors. Am J Transl Res. 2023-01-01; 15 (5): 3468-3475. PMID: 37303624;
8. King, CA, Perez-Alvarez, IM, Bartholomew, AJ, Bozzuto, L, Griffith, K, Sosin, M, Thibodeau, R, Gopwani, S, Myers, J, Fan, KL, Tousimis, EA. Opioid-free anesthesia for patients undergoing mastectomy: A matched comparison. BREAST J. 2020-09-01; 26 (9): 1742-1747. doi: 10.1111/tbj.13999. PMID: 32767477;
9. Mulier, H, De Frene, B, Benmeridja, L, et al. Impact of opioid-free anesthesia on complications after deep inferior epigastric perforator flap surgery: A retrospective cohort study. J PLAST RECONSTR AES. 2020; 74 (3): 504-511. doi: 10.1016/j.bjps.2020.09.004
10. Saad-Boutry, M, Carton, M, Ezzili, C, et al. Opioid-free versus opioid-based anaesthesia for free-flap reconstruction surgery of the breast: protocol for a phase III, multicentre, randomised controlled study. BMJ Open. 2025; 15 (2): e070021. doi: 10.1136/bmjopen-2022-070021
11. Hong, B, Bang, S, Chung, W, Yoo, S, Chung, J, Kim, S. Multimodal analgesia with multiple intermittent doses of erector spinae plane block through a catheter after total mastectomy: a retrospective observational study. KOREAN J PAIN. 2019-07-01; 32 (3): 206-214. doi: 10.3344/kjp.2019.32.3.206. PMID: 31257829;
12. Khan, JS, Gilron, I, Devereaux, PJ, Clarke, H, Ayach, N, Tomlinson, G, Quan, ML, Ladha, KS, Choi, S, Munro, A, Brull, R, Lim, DW, Avramescu, S, Richebé, P, Hodgson, N, Paul, J, McIsaac, DI, Derzi, S, Zbitnew, GL, Easson, AM, Siddiqui, NT, Miles, SJ, Karkouti, K. Prevention of persistent pain with lidocaine infusions in breast cancer surgery (PLAN): study protocol for a multicenter randomized controlled trial. Trials. 2024-05-22; 25 (1): 337. doi: 10.1186/s13063-024-08151-4. PMID: 38773653;
13. Wang, J, Doan, LV, Axelrod, D, Rotrosen, J, Wang, B, Park, HG, Edwards, RR, Curatolo, M, Jackman, C, Perez, R. Optimizing the use of ketamine to reduce chronic postsurgical pain in women undergoing mastectomy for oncologic indication: study protocol for the KALPAS multicenter randomized controlled trial. Trials. 2024-01-19; 25 (1): 67. doi: 10.1186/s13063-023-07884-y. PMID: 38243266;
14. Morioka, H, Kamiya, Y, Yoshida, T, Baba, H. Pectoral nerve block combined with general anesthesia for breast cancer surgery: a retrospective comparison. JA Clin Rep. 2015-01-01; 1 (1): 15. doi: 10.1186/s40981-015-0018-1. PMID: 29497647;

**Surgery type mismatch:**

1. You, X, Jiang, G. Effect of Ropivacaine Intercostal Nerve Block Combined with Patient Controlled Intravenous Analgesia on Postoperative Analgesia after Breast Augmentation. AESTHET PLAST SURG. 2024-10-01; 48 (20): 4137-4141. doi: 10.1007/s00266-024-03856-y. PMID: 38388796;
2. Ciftci, B, Ekinci, M, Celik, EC, Karaaslan, P, Tukac, İC. Ultrasound-guided pectoral nerve block for pain control after breast augmentation: a randomized clinical study. Braz J Anesthesiol. 2021-01-01; 71 (1): 44-49. doi: 10.1016/j.bjane.2020.12.004. PMID: 33712252;
3. Clary, Z, Nazir, N, Butterworth, J. Transversus Abdominis Plane Block With Liposomal Bupivacaine Versus Thoracic Epidural for Postoperative Analgesia After Deep Inferior Epigastric Artery Perforator Flap-Based Breast Reconstruction. ANN PLAS SURG. 2020-12-01; 85 (6): e24-e26. doi: 10.1097/SAP.0000000000002423. PMID: 33170580;
4. Hammond, DC. Bilateral Ultrasound-Guided Erector Spinae Plane Block for Perioperative Analgesia in Breast Reduction Surgery: A Prospective Randomized and Controlled Trial. AESTHET PLAST SURG. 2023-08-01; 47 (4): 1289-1290. doi: 10.1007/s00266-023-03336-9. PMID: 37145317;
5. DiGiorgi, M, Carangelo, M, Scranton, R. Transversus Abdominis Plane Blocks with Single-Dose Liposomal Bupivacaine in Conjunction with a Nonnarcotic Pain Regimen Help Reduce Length of Stay following Abdominally Based Microsurgical Breast Reconstruction. PLAST RECONSTR SURG. 2018-07-01; 142 (1): 94e. doi: 10.1097/PRS.0000000000004480. PMID: 29742651;
