# Supplementary figures and images for: Opioid-sparing anesthesia versus opioid-free anesthesia for postoperative recovery quality in breast cancer surgery patients: A systematic review and Bayesian network meta-analysis
Source: PLoS One. 2025 Oct 24;20(10):e0334614. doi: 10.1371/journal.pone.0334614 (PMC12551851; doi:10.1371/journal.pone.0334614)

**Quality of Evidence Assessment**


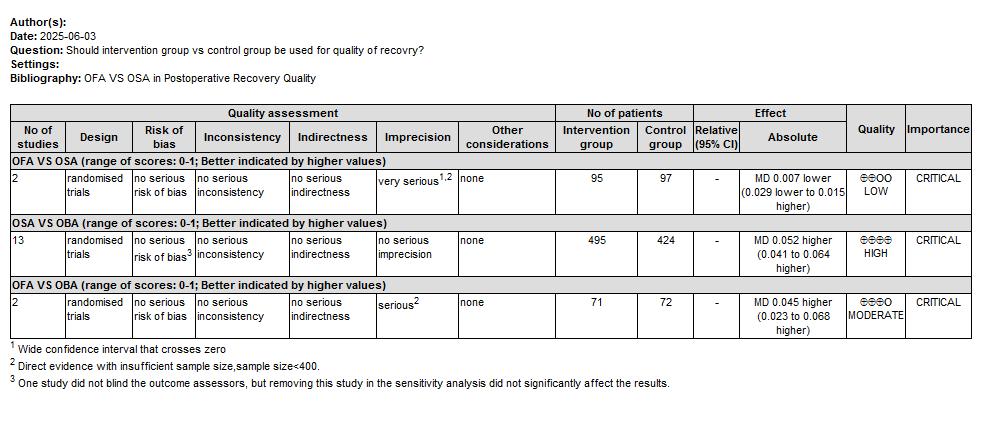


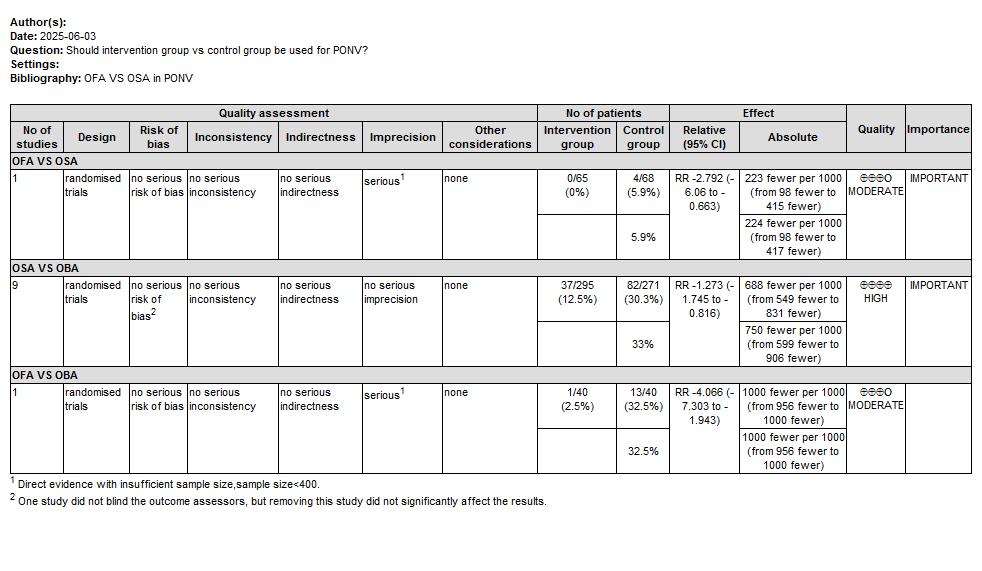


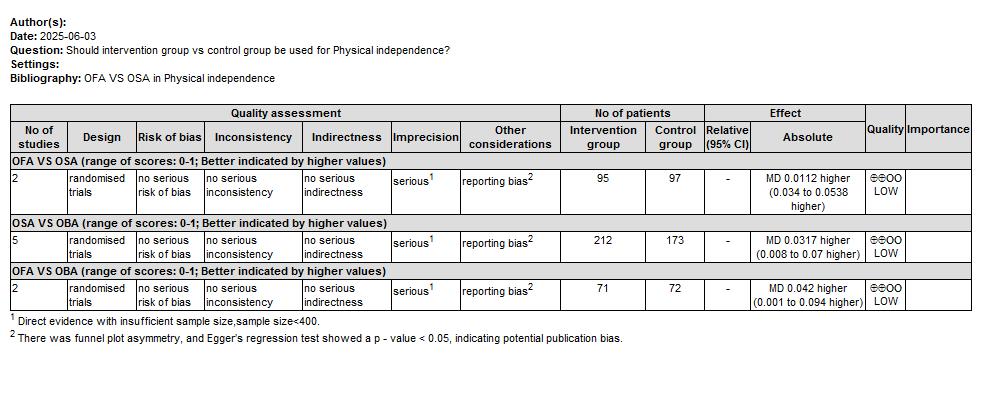


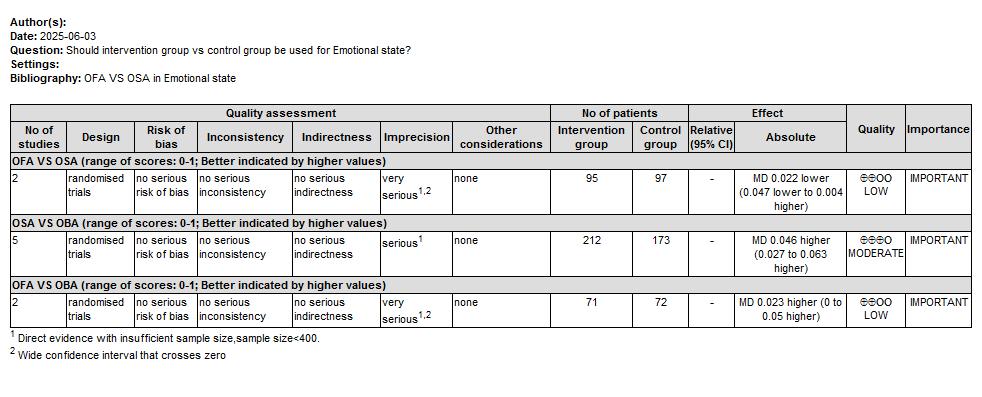


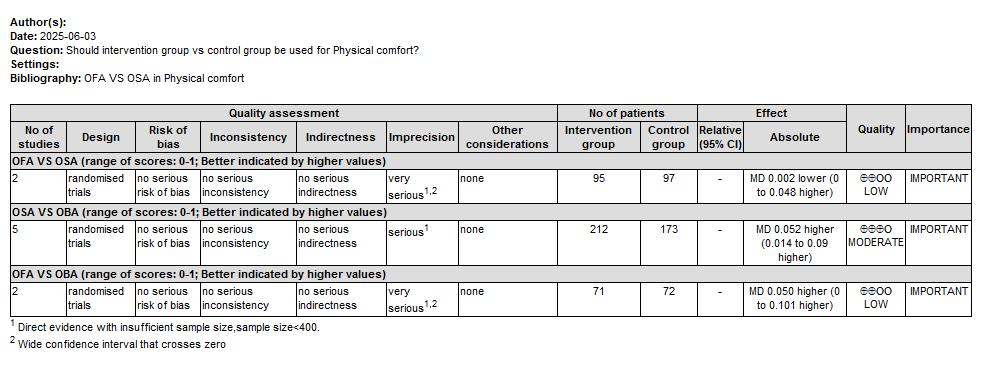


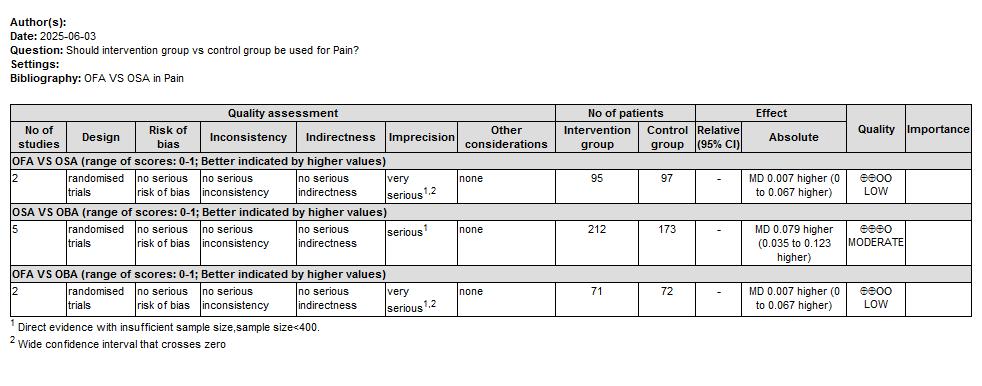


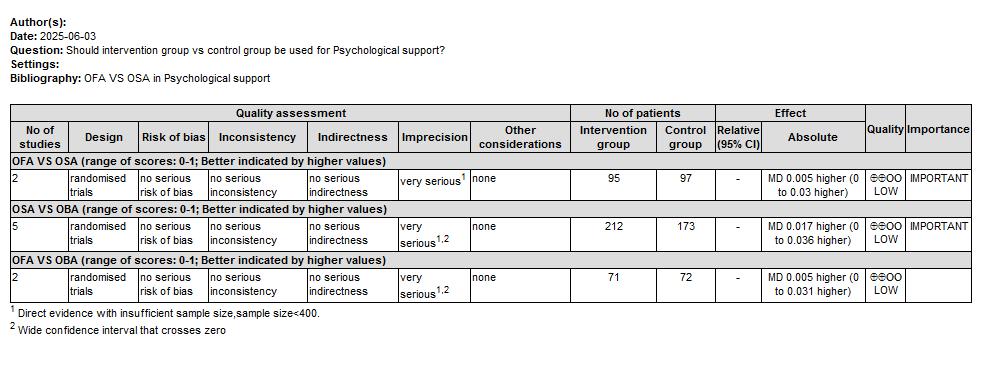

Supplement: S3 Text — GRADE evaluation for primary/secondary outcomes. (DOCX) [file pone.0334614.s004.docx]
