# Supplementary material for: Opioid-sparing anesthesia versus opioid-free anesthesia for postoperative recovery quality in breast cancer surgery patients: A systematic review and Bayesian network meta-analysis
Source: PLoS One. 2025 Oct 24;20(10):e0334614. doi: 10.1371/journal.pone.0334614 (PMC12551851; doi:10.1371/journal.pone.0334614)

**Funnel plots and the results of Egger’s regression tests**

QOR score：Egger’s regression tests, p = 0.9141

**
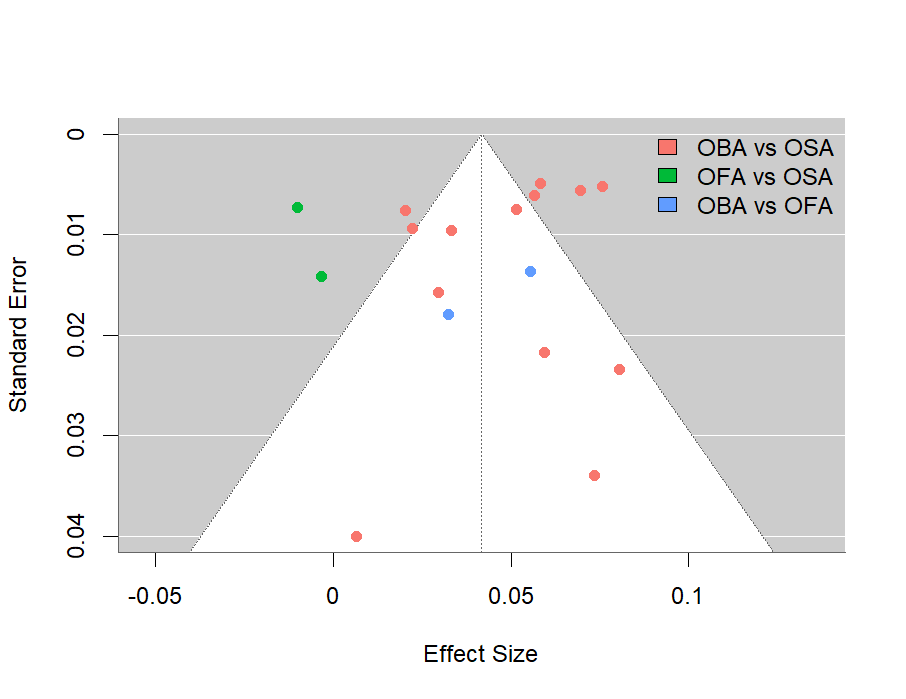
**

PONV：Egger’s regression tests, p = 0.3491

**
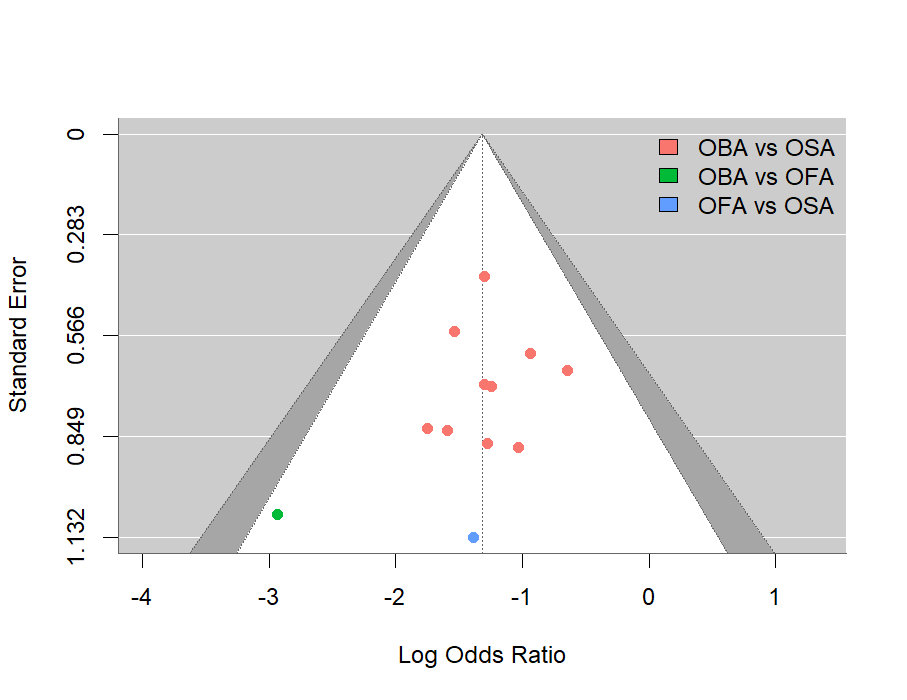
**

physical independence：Egger’s regression tests,p = 0.0233


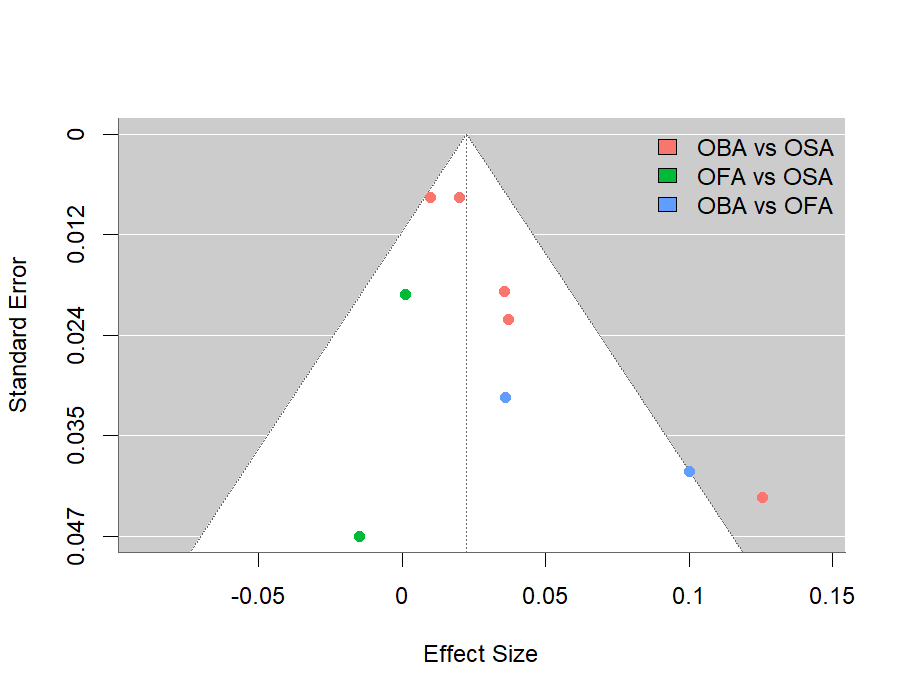


emotional state: Egger’s regression tests,p = 0.5662


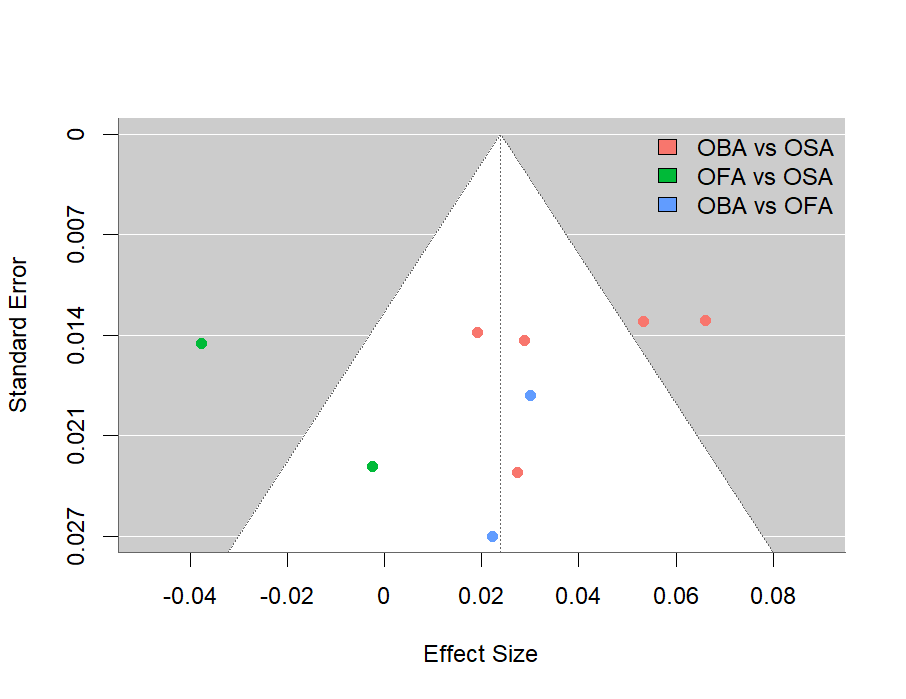


physical comfort：Egger’s regression tests, p = 0.5242


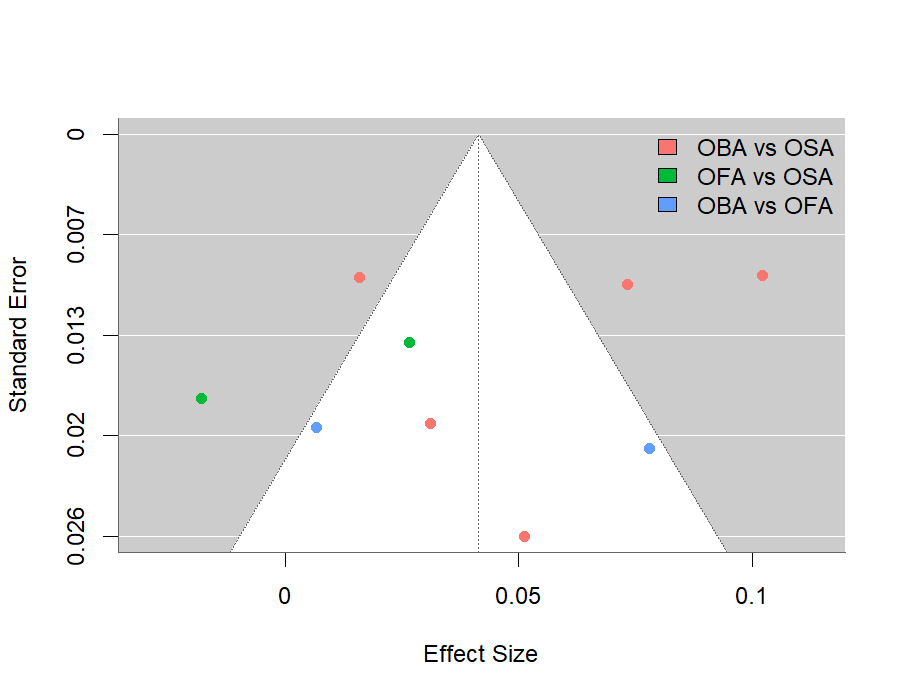


Pain: Egger’s regression tests, p = 0.5106


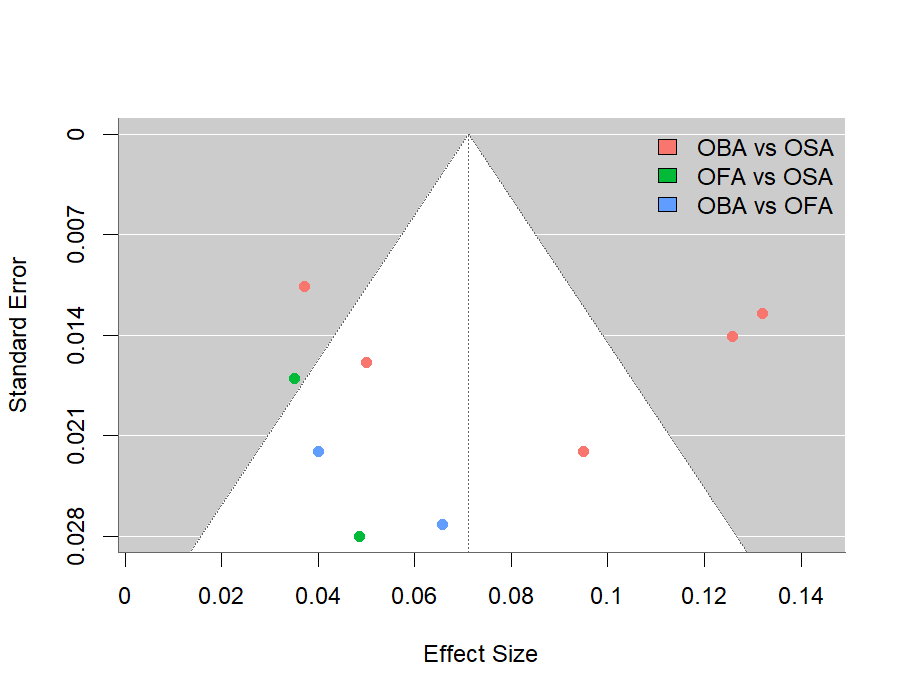


psychological support：Egger’s regression tests, p = 0.5181


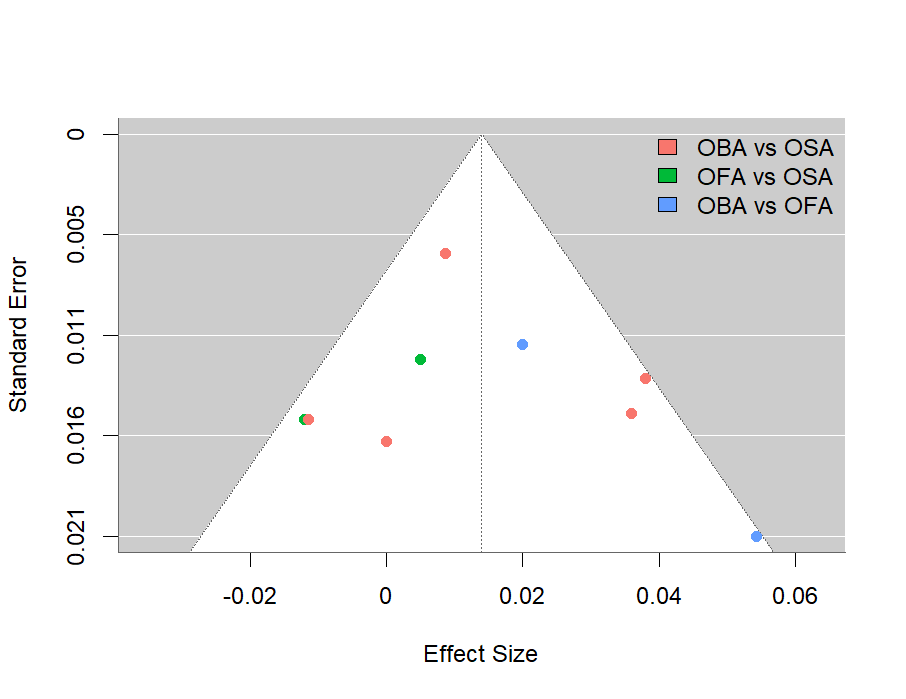

Supplement: S4 Text — Funnel plots and the results of Egger’s regression tests. (DOCX) [file pone.0334614.s005.docx]
