# Supplementary material for: Opioid-sparing anesthesia versus opioid-free anesthesia for postoperative recovery quality in breast cancer surgery patients: A systematic review and Bayesian network meta-analysis
Source: PLoS One. 2025 Oct 24;20(10):e0334614. doi: 10.1371/journal.pone.0334614 (PMC12551851; doi:10.1371/journal.pone.0334614)
Supplement: S5 Text — Results of Gelman-Rubin diagnostic, effective sample size, and Node-Splitting inconsistency test. (DOCX) [file pone.0334614.s006.docx]

**Results of Gelman-Rubin Diagnostic, Effective Sample Size, and Node-Splitting Inconsistency Test**

**QOR score：**

Rhat:

Point est. Upper C.I.

d.OBA.OFA 0.9999221 1.0000226

d.OBA.OSA 0.9999085 0.9999651

sd.d 1.0001424 1.0001679

ESS:

d.OBA.OFA d.OBA.OSA sd.d

20238.26 20000.00 20031.27

Node-splitting analysis of inconsistency：

comparison p.value CrI

1 d.OSA.OBA 0.9109

2 -> direct -0.050 (-0.063, -0.036)

3 -> indirect -0.053 (-0.10, -0.0046)

4 -> network -0.050 (-0.062, -0.037)

5 d.OBA.OFA 0.9154

6 -> direct 0.045 (0.0095, 0.081)

7 -> indirect 0.043 (0.0077, 0.078)

8 -> network 0.044 (0.020, 0.068)

9 d.OSA.OFA 0.9008

10 -> direct -0.0075 (-0.039, 0.025)

11 -> indirect -0.0045 (-0.042, 0.034)

12 -> network -0.0062 (-0.030, 0.018)

**PONV****：**

Rhat:

Point est. Upper C.I.

d.OBA.OFA 1.001177 1.002949

d.OBA.OSA 1.000418 1.000559

sd.d 1.000617 1.001778

ESS:

d.OBA.OFA d.OBA.OSA sd.d

4313.961 6947.116 5944.303

Node-splitting analysis of inconsistency：

comparison p.value CrI

1 d.OSA.OBA 0.8590

2 -> direct 1.3 (0.77, 1.8)

3 -> indirect 1.6 (-2.4, 5.7)

4 -> network 1.3 (0.78, 1.8)

5 d.OBA.OFA 0.8711

6 -> direct -3.3 (-6.7, -1.2)

7 -> indirect -3. (-6.5, -0.63)

8 -> network -3.0 (-5.0, -1.5)

9 d.OSA.OFA 0.8694

10 -> direct -1.7 (-5.4, 0.58)

11 -> indirect -2.0 (-5.5, 0.099)

12 -> network -1.7 (-3.8, -0.20)

**Physical independence****：**

Rhat:

Point est. Upper C.I.

d.OBA.OFA 1.000292 1.000446

d.OBA.OSA 1.000801 1.001796

sd.d 1.001367 1.004402

ESS:

d.OBA.OFA d.OBA.OSA sd.d

15238.04 14613.77 9704.58

Node-splitting analysis of inconsistency：

comparison p.value CrI

1 d.OSA.OBA 0.3687

2 -> direct -0.026 (-0.069, -0.0025)

3 -> indirect -0.064 (-0.15, 0.019)

4 -> network -0.029 (-0.068, -0.0083)

5 d.OBA.OFA 0.3547

6 -> direct 0.062 (0.0011, 0.13)

7 -> indirect 0.024 (-0.035, 0.097)

8 -> network 0.041 (0.0014, 0.091)

9 d.OSA.OFA 0.3636

10 -> direct -0.0023 (-0.063, 0.055)

11 -> indirect 0.035 (-0.041, 0.10)

12 -> network 0.011 (-0.034, 0.053)

**Emotional state****：**

Rhat:

Point est. Upper C.I.

d.OBA.OFA 1.000133 1.000436

d.OBA.OSA 1.000265 1.000749

sd.d 1.000359 1.000936

ESS:

d.OBA.OFA d.OBA.OSA sd.d

18339.12 19083.55 11743.03

Node-splitting analysis of inconsistency：

comparison p.value CrI

1 d.OSA.OBA 0.6739

2 -> direct -0.041 (-0.063, -0.016)

3 -> indirect -0.052 (-0.11, 0.0087)

4 -> network -0.042 (-0.061, -0.021)

5 d.OBA.OFA 0.6681

6 -> direct 0.028 (-0.016, 0.070)

7 -> indirect 0.015 (-0.029, 0.062)

8 -> network 0.022 (-0.0073, 0.052)

9 d.OSA.OFA 0.6915

10 -> direct -0.025 (-0.062, 0.017)

11 -> indirect -0.013 (-0.062, 0.036)

12 -> network -0.020 (-0.048, 0.0094)

**Physical comfort：**

Rhat:

Point est. Upper C.I.

d.OBA.OFA 1.000144 1.000579

d.OBA.OSA 1.000419 1.001255

sd.d 1.000037 1.000123

ESS:

d.OBA.OFA d.OBA.OSA sd.d

20311.36 21207.85 20000.00

Node-splitting analysis of inconsistency：

comparison p.value CrI

1 d.OSA.OBA 0.7029

2 -> direct -0.056 (-0.10, -0.010)

3 -> indirect -0.037 (-0.14, 0.064)

4 -> network -0.053 (-0.090, -0.015)

5 d.OBA.OFA 0.6890

6 -> direct 0.042 (-0.031, 0.11)

7 -> indirect 0.061 (-0.024, 0.14)

8 -> network 0.050 (-0.0020, 0.10)

9 d.OSA.OFA 0.6966

10 -> direct 0.0057 (-0.067, 0.077)

11 -> indirect -0.014 (-0.097, 0.073)

12 -> network -0.0023 (-0.054, 0.049)

**Pain****：**

Rhat:

Point est. Upper C.I.

d.OBA.OFA 1.0001442 1.000323

d.OBA.OSA 1.0000430 1.000262

sd.d 0.9999713 1.000235

ESS:

d.OBA.OFA d.OBA.OSA sd.d

18959.01 19999.97 19446.41

Node-splitting analysis of inconsistency：

comparison p.value CrI

1 d.OSA.OBA 0.1751

2 -> direct -0.088 (-0.14, -0.041)

3 -> indirect -0.011 (-0.12, 0.099)

4 -> network -0.076 (-0.12, -0.030)

5 d.OBA.OFA 0.1768

6 -> direct 0.052 (-0.026, 0.13)

7 -> indirect 0.13 (0.039, 0.22)

8 -> network 0.086 (0.021, 0.15)

9 d.OSA.OFA 0.1722

10 -> direct 0.041 (-0.035, 0.12)

11 -> indirect -0.036 (-0.13, 0.057)

12 -> network 0.0089 (-0.054, 0.070)

**Psychological support：**

Rhat:

Point est. Upper C.I.

d.OBA.OFA 1.000172 1.000365

d.OBA.OSA 1.000236 1.000422

sd.d 1.000831 1.002102

ESS:

d.OBA.OFA d.OBA.OSA sd.d

17693.82 18944.50 13378.63

Node-splitting analysis of inconsistency：

comparison p.value CrI

1 d.OSA.OBA 0.3918

2 -> direct -0.014 (-0.037, 0.0076)

3 -> indirect -0.034 (-0.090, 0.014)

4 -> network -0.017 (-0.037, 0.00094)

5 d.OBA.OFA 0.4018

6 -> direct 0.032 (-0.0031, 0.072)

7 -> indirect 0.012 (-0.031, 0.053)

8 -> network 0.022 (-0.0015, 0.050)

9 d.OSA.OFA 0.4056

10 -> direct -0.0022 (-0.038, 0.033)

11 -> indirect 0.017 (-0.023, 0.062)

12 -> network 0.0055 (-0.019, 0.032)
