# Supplementary material for: Opioid-sparing anesthesia versus opioid-free anesthesia for postoperative recovery quality in breast cancer surgery patients: A systematic review and Bayesian network meta-analysis
Source: PLoS One. 2025 Oct 24;20(10):e0334614. doi: 10.1371/journal.pone.0334614 (PMC12551851; doi:10.1371/journal.pone.0334614)
Supplement: S6 Text — Heterogeneity and comparison of prior and posterior treatment effects. (DOCX) [file pone.0334614.s007.docx]

**Heterogeneity and Comparison of Prior and Posterior Treatment Effects**

**QOR score：**

Default:

Mean SD Q2.5 Median Q97.5

2.5% 0.01831998 0.005043438 0.01059262 0.01762379 0.03032908

Inverse-Gamma:

Mean SD Q2.5 Median Q97.5

2.5% 0.01981595 0.005630759 0.01122477 0.01894539 0.03308726

Semi-Normal:

Mean SD Q2.5 Median Q97.5

2.5% 0.01825954 0.005021245 0.01070193 0.017562 0.03039418


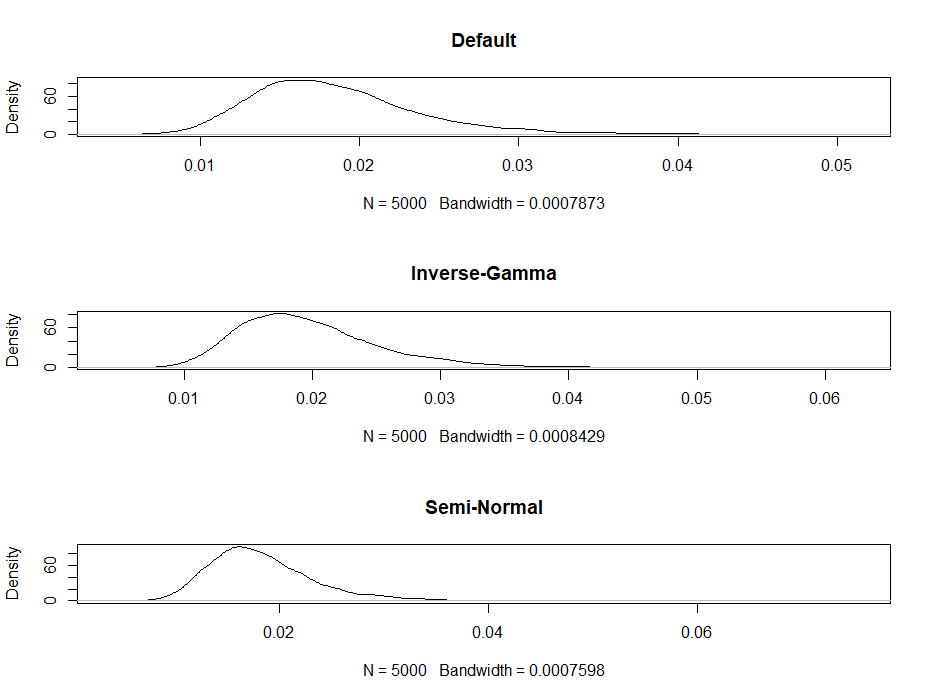


Default DIC:

Dbar pD DIC data points

32.73477 28.94538 61.68015 34.00000

Inverse-Gamma DIC:

Dbar pD DIC data points

32.56813 29.27297 61.84110 34.00000

Semi-Normal DIC:

Dbar pD DIC data points

32.82887 28.99467 61.82354 34.00000

Default:

Mean 2.5%.2.5% 97.5%.97.5%

OSA vs OBA 0.05007103 0.03769959 0.06189597

OFA vs OBA 0.04380916 0.01994081 0.06733520

OSA vs OFA 0.00626187 -0.01750456 0.02981065

check.names 0.00000000 0.00000000 0.00000000

Half-Cauchy:

Mean 2.5%.2.5% 97.5%.97.5%

OSA vs OBA 0.050047441 0.03764524 0.06219286

OFA vs OBA 0.043930043 0.01994408 0.06798532

OSA vs OFA 0.006117399 -0.01746838 0.02972726

check.names 0.000000000 0.00000000 0.00000000


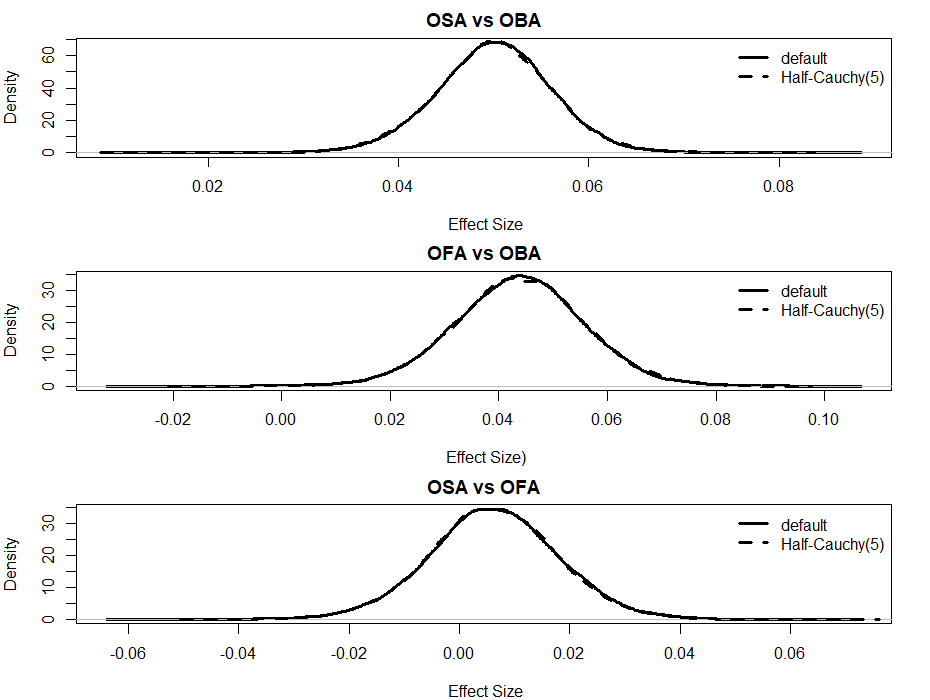


Default DIC:

Dbar pD DIC data points

32.81964 29.02941 61.84905 34.00000

Half-Cauchy DIC:

Dbar pD DIC data points

32.84080 29.06423 61.90503 34.00000

**PONV：**

Default:

Mean SD Q2.5 Median Q97.5

2.5% 0.2749973 0.2320736 0.009155967 0.2201147 0.8620286

Inverse-Gamma:

Mean SD Q2.5 Median Q97.5

2.5% 0.4665001 0.28836 0.07441924 0.4111814 1.187264

Semi-Normal:

Mean SD Q2.5 Median Q97.5

2.5% 0.2559725 0.20818 0.008695878 0.2087094 0.7718781


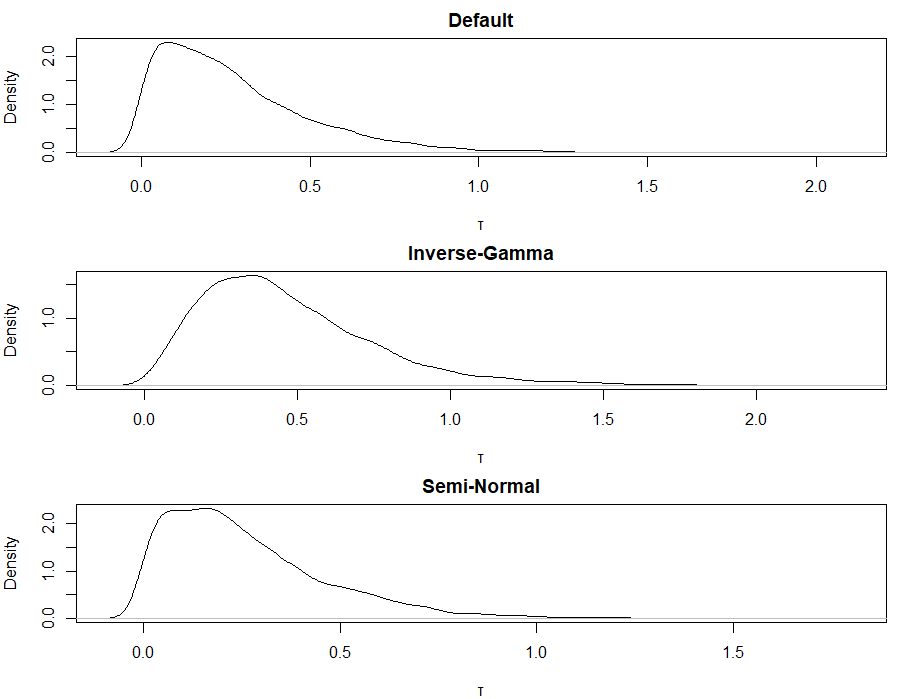


Model DIC pD

1 Default Prior 30.59068 14.57784

2 Inverse-Gamma Prior 32.61897 15.80113

3 Half-Normal Prior 30.53449 14.52424

Default:

Mean 2.5%.2.5% 97.5%.97.5%

OSA vs OBA -1.293636 -1.818292 -0.7867088

OFA vs OBA -3.076874 -5.141871 -1.4997810

OFA vs OSA -1.783239 -3.862025 -0.1632728

check.names 0.000000 0.000000 0.0000000

Half-Cauchy:

Mean 2.5%.2.5% 97.5%.97.5%

OSA vs OBA -1.298052 -1.840342 -0.7806499

OFA vs OBA -3.078918 -5.017474 -1.5201564

OFA vs OSA -1.780866 -3.714069 -0.1740947

check.names 0.000000 0.000000 0.0000000


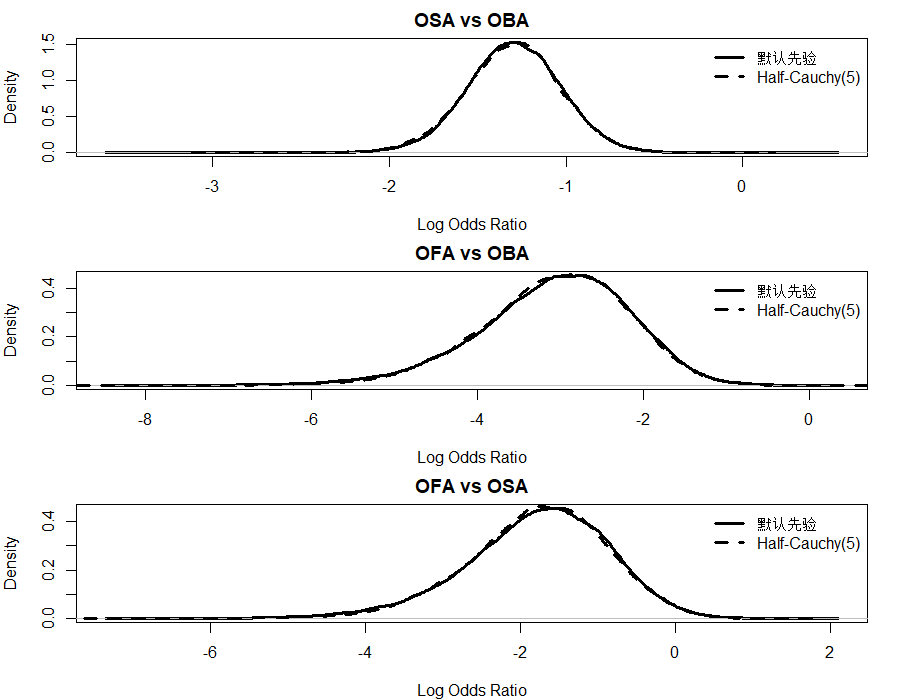


Model DIC pD

1 Default Prior 30.67990 14.61878

2 Half-Cauchy(0,5) Prior 30.64521 14.60531

**Physical independence：**

Default:

Mean SD Q2.5 Median Q97.5

2.5% 0.02282637 0.01707566 0.001285031 0.01928239 0.06651349

Inverse-Gamma:

Mean SD Q2.5 Median Q97.5

2.5% 0.03601442 0.02292141 0.007142101 0.03104059 0.09444465

Semi-Normal:

Mean SD Q2.5 Median Q97.5

2.5% 0.02304373 0.0178809 0.0009786412 0.01947392 0.06834363


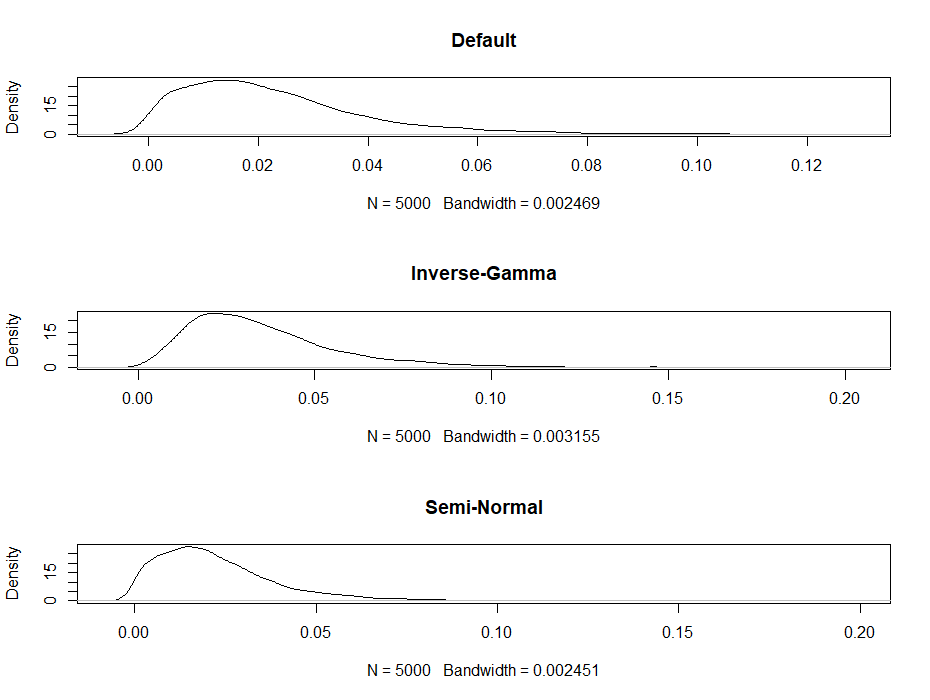


Default DIC:

Dbar pD DIC data points

19.77045 14.68570 34.45615 18.00000

Inverse-Gamma DIC:

Dbar pD DIC data points

18.41437 15.66898 34.08335 18.00000

Semi-Normal DIC:

Dbar pD DIC data points

19.77752 14.72027 34.49779 18.00000

Default:

Mean 2.5%.2.5% 97.5%.97.5%

OSA vs OBA 0.03148063 0.008573174 0.06910275

OFA vs OBA 0.04295279 0.002332808 0.09411376

OSA vs OFA -0.01147216 -0.054819325 0.03270039

check.names 0.00000000 0.000000000 0.00000000

Half-Cauchy:

Mean 2.5%.2.5% 97.5%.97.5%

OSA vs OBA 0.03142417 0.008694528 0.06770101

OFA vs OBA 0.04274137 0.001062204 0.09231832

OSA vs OFA -0.01131720 -0.053238174 0.03328394

check.names 0.00000000 0.000000000 0.00000000


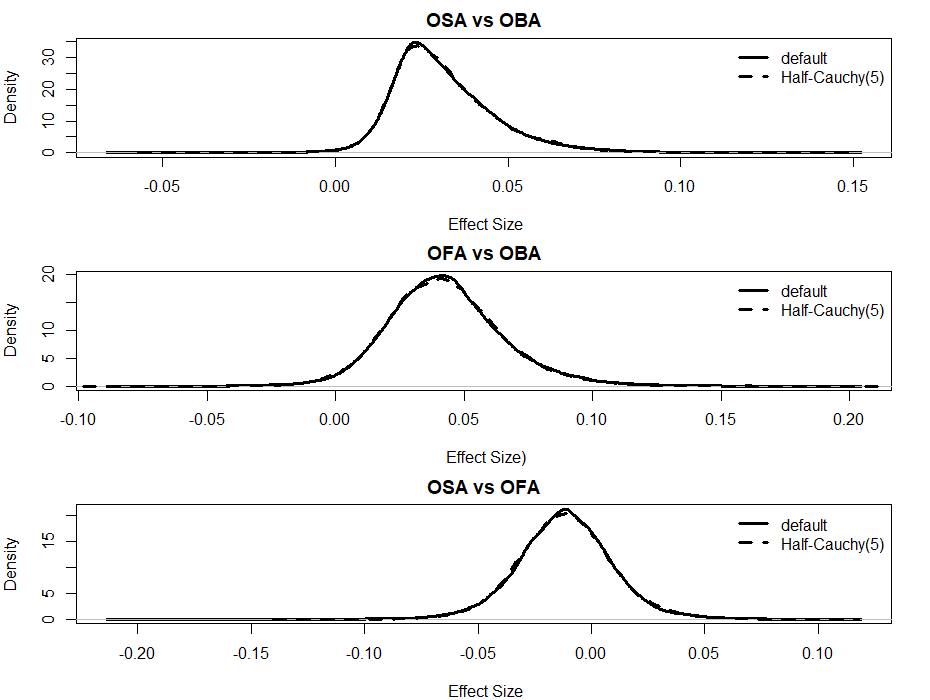


Default DIC:

Dbar pD DIC data points

19.73595 14.68602 34.42197 18.00000

Half-Cauchy DIC:

Dbar pD DIC data points

19.77826 14.67823 34.45649 18.00000

**Emotional state：**

Default:

Mean SD Q2.5 Median Q97.5

2.5% 0.01591497 0.009964185 0.001062489 0.01468467 0.03974429

Inverse-Gamma:

Mean SD Q2.5 Median Q97.5

2.5% 0.02329793 0.01291752 0.005648256 0.02085178 0.05442731

Semi-Normal:

Mean SD Q2.5 Median Q97.5

2.5% 0.0162617 0.01077893 0.0009838033 0.01467796 0.0422905


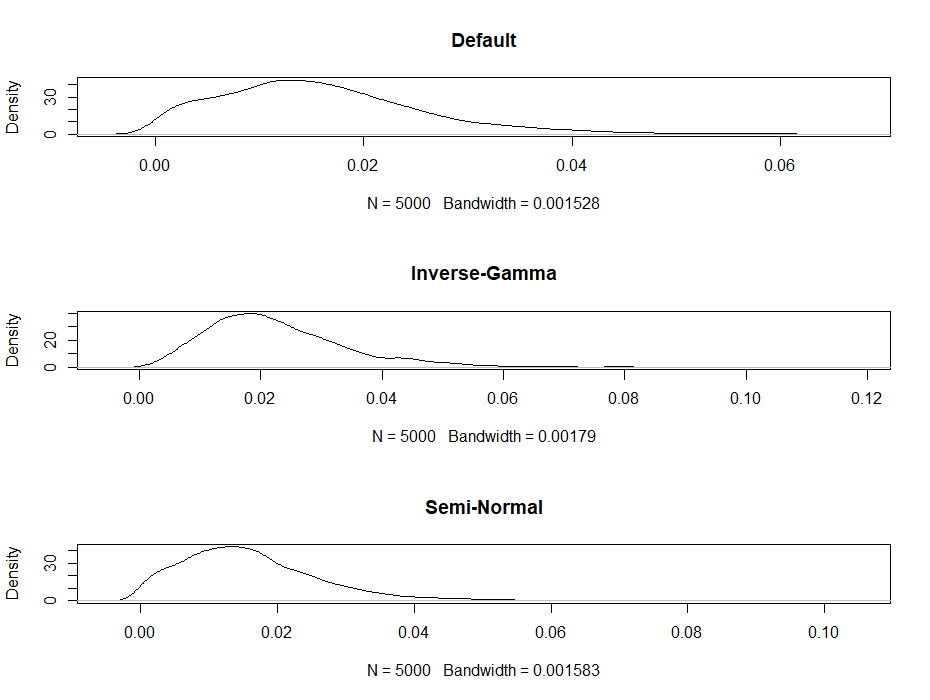


Default DIC:

Dbar pD DIC data points

17.49483 14.62203 32.11685 18.00000

Inverse-Gamma DIC:

Dbar pD DIC data points

16.88906 15.45594 32.34500 18.00000

Semi-Normal DIC:

Dbar pD DIC data points

17.54179 14.66745 32.20924 18.00000

Default:

Mean 2.5%.2.5% 97.5%.97.5%

OSA vs OBA 0.04205634 0.021389541 0.06179039

OFA vs OBA 0.02164003 -0.007422029 0.05133057

OSA vs OFA 0.02041631 -0.009528620 0.04830737

check.names 0.00000000 0.000000000 0.00000000

Half-Cauchy:

Mean 2.5%.2.5% 97.5%.97.5%

OSA vs OBA 0.04199007 0.021194790 0.06133388

OFA vs OBA 0.02174389 -0.007175520 0.05164412

OSA vs OFA 0.02024617 -0.009603377 0.04829736

check.names 0.00000000 0.000000000 0.00000000


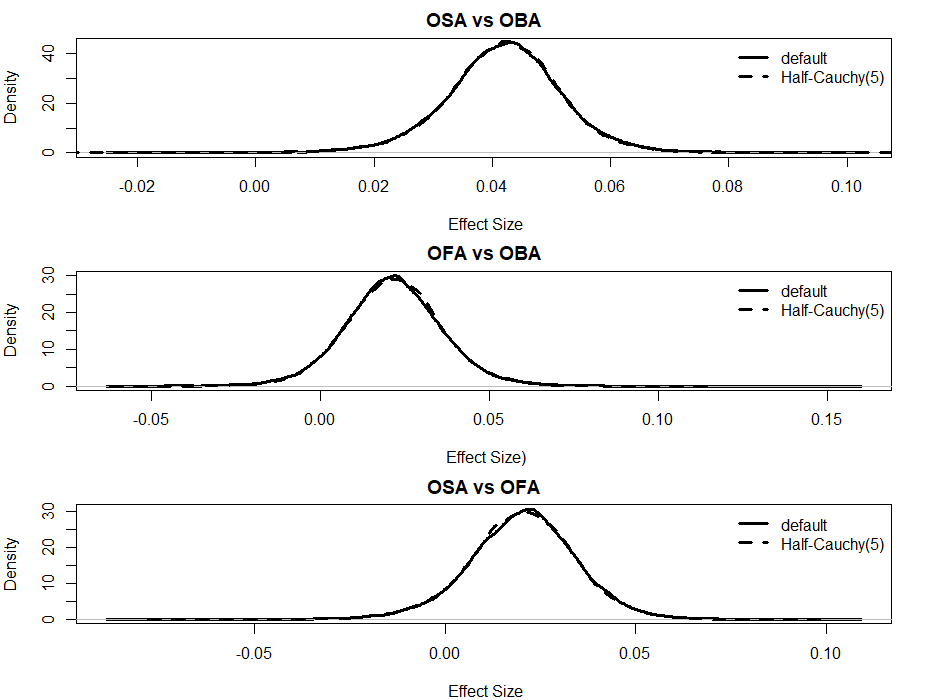


Default DIC:

Dbar pD DIC data points

17.49133 14.65226 32.14359 18.00000

Half-Cauchy DIC:

Dbar pD DIC data points

17.49119 14.59759 32.08878 18.00000

**Physical comfort：**

Default:

Mean SD Q2.5 Median Q97.5

2.5% 0.04090903 0.01463243 0.02063438 0.03797236 0.07855771

Inverse-Gamma:

Mean SD Q2.5 Median Q97.5

2.5% 0.04870666 0.02313304 0.02248195 0.0431473 0.1064969

Semi-Normal:

Mean SD Q2.5 Median Q97.5

2.5% 0.04140017 0.01593971 0.02082953 0.03776005 0.0832469


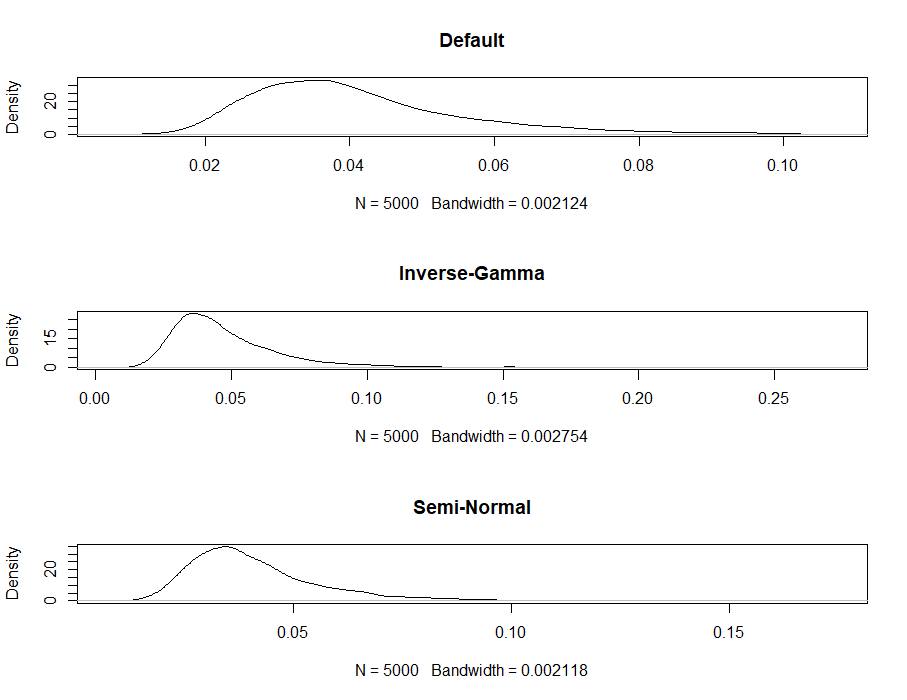


Default DIC:

Dbar pD DIC data points

17.70135 16.97715 34.67849 18.00000

Inverse-Gamma DIC:

Dbar pD DIC data points

17.69073 17.21244 34.90317 18.00000

Semi-Normal DIC:

Dbar pD DIC data points

17.75785 17.05381 34.81166 18.00000

Default:

Mean 2.5%.2.5% 97.5%.97.5%

OSA vs OBA 0.052763405 0.014889541 0.08982888

OFA vs OBA 0.050198081 -0.001005568 0.09998486

OSA vs OFA 0.002565324 -0.048000025 0.05282497

check.names 0.000000000 0.000000000 0.00000000

Half-Cauchy:

Mean 2.5%.2.5% 97.5%.97.5%

OSA vs OBA 0.052663032 0.014351344 0.09069051

OFA vs OBA 0.050174397 -0.002167025 0.10141124

OSA vs OFA 0.002488635 -0.047782934 0.05278585

check.names 0.000000000 0.000000000 0.00000000


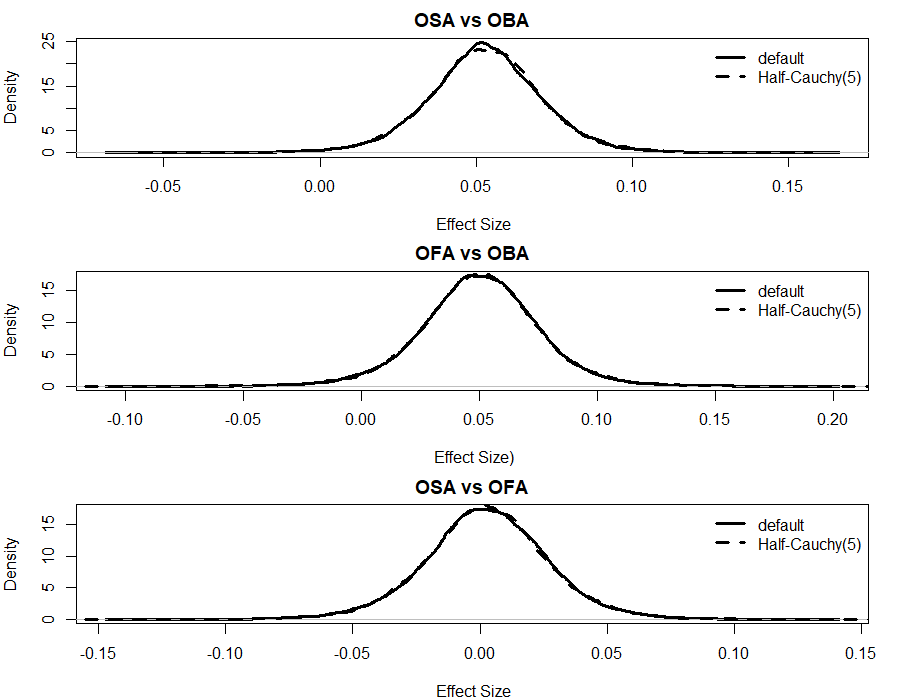


Default DIC:

Dbar pD DIC data points

17.73101 17.01109 34.74210 18.00000

Half-Cauchy DIC:

Dbar pD DIC data points

17.79637 17.07957 34.87594 18.00000

**Pain：**

Default:

Mean SD Q2.5 Median Q97.5

2.5% 0.04959276 0.01778287 0.02493869 0.0459204 0.09524206

Inverse-Gamma:

Mean SD Q2.5 Median Q97.5

2.5% 0.0590973 0.03028184 0.02755931 0.05257319 0.1260613

Semi-Normal:

Mean SD Q2.5 Median Q97.5

2.5% 0.05038943 0.02015185 0.02480306 0.04601802 0.1028005


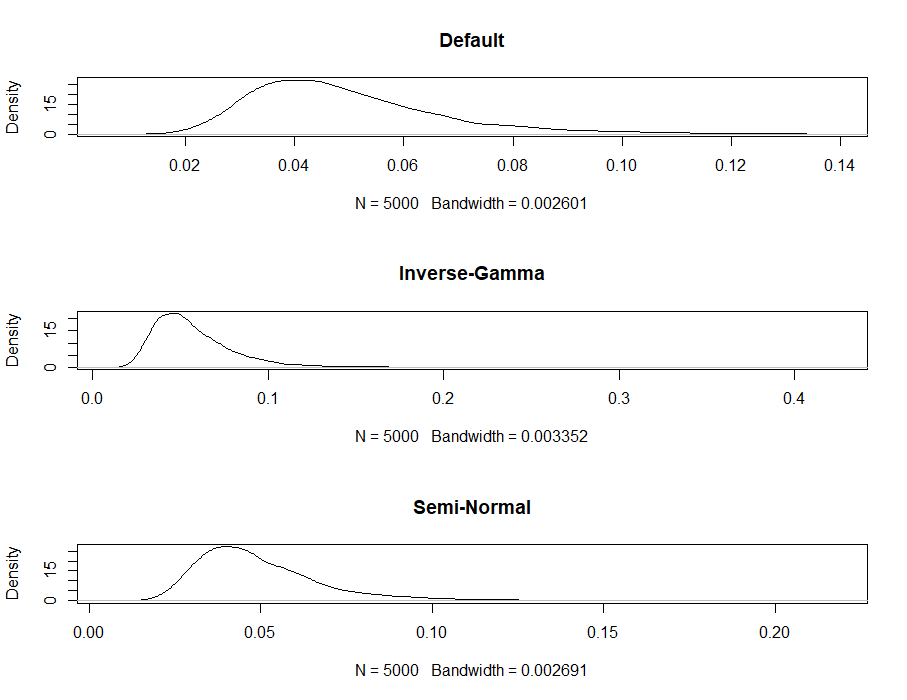


Default DIC:

Dbar pD DIC data points

17.95144 17.16864 35.12008 18.00000

Inverse-Gamma DIC:

Dbar pD DIC data points

17.80724 17.27725 35.08449 18.00000

Semi-Normal DIC:

Dbar pD DIC data points

17.88799 17.09638 34.98437 18.00000

Default:

Mean 2.5%.2.5% 97.5%.97.5%

OSA vs OBA 0.076371724 0.03041680 0.12176702

OFA vs OBA 0.085605233 0.02282976 0.14766261

OSA vs OFA -0.009233509 -0.07165603 0.05380639

check.names 0.000000000 0.00000000 0.00000000

Half-Cauchy:

Mean 2.5%.2.5% 97.5%.97.5%

OSA vs OBA 0.076386450 0.03036460 0.12146268

OFA vs OBA 0.085615669 0.02262249 0.14749261

OSA vs OFA -0.009229219 -0.07068515 0.05296444

check.names 0.000000000 0.00000000 0.00000000


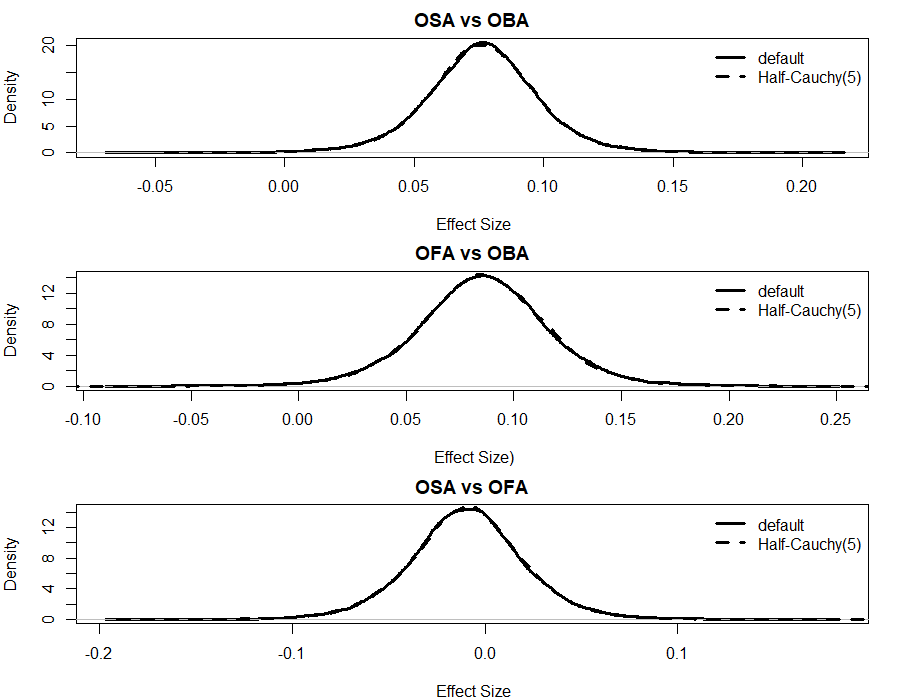


Default DIC:

Dbar pD DIC data points

17.82682 17.05015 34.87698 18.00000

Half-Cauchy DIC:

Dbar pD DIC data points

17.96405 17.16889 35.13294 18.00000

**Psychological support：**

Default:

Mean SD Q2.5 Median Q97.5

2.5% 0.0159388 0.009816091 0.001275781 0.01441258 0.03945785

Inverse-Gamma:

Mean SD Q2.5 Median Q97.5

2.5% 0.0234862 0.01305722 0.005981083 0.02087468 0.05752532

Semi-Normal:

Mean SD Q2.5 Median Q97.5

2.5% 0.01632952 0.01069361 0.00108743 0.01475466 0.04175369


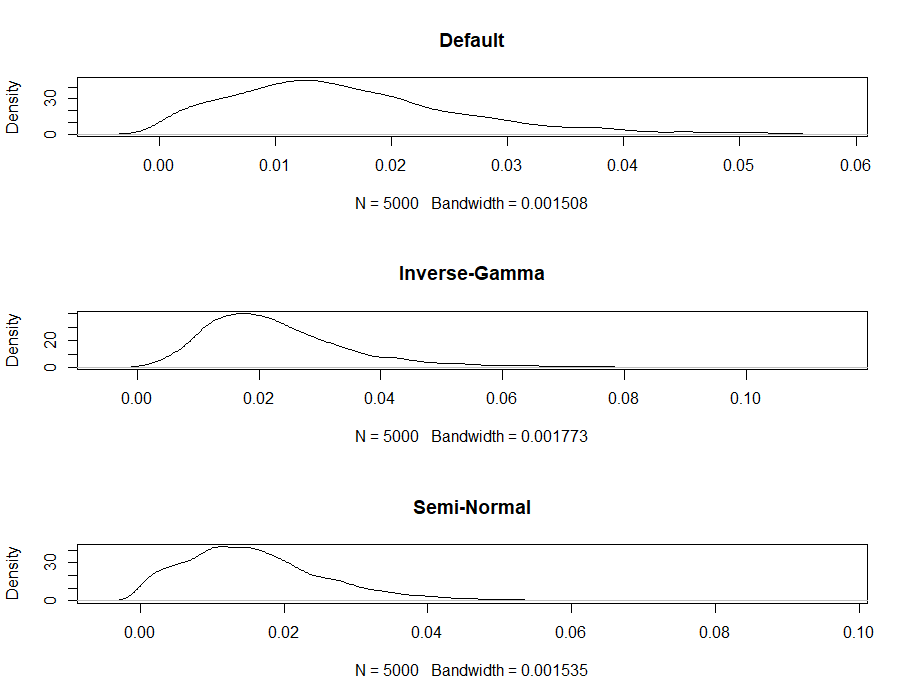


Default DIC:

Dbar pD DIC data points

19.41746 15.47042 34.88788 18.00000

Inverse-Gamma DIC:

Dbar pD DIC data points

18.20817 16.22031 34.42848 18.00000

Semi-Normal DIC:

Dbar pD DIC data points

19.36176 15.42926 34.79103 18.00000

Default:

Mean 2.5%.2.5% 97.5%.97.5%

OSA vs OBA 0.017202499 -0.0007392071 0.03670128

OFA vs OBA 0.022677931 -0.0018149740 0.04990194

OSA vs OFA -0.005475431 -0.0313446327 0.01952808

check.names 0.000000000 0.0000000000 0.00000000

Half-Cauchy :

Mean 2.5%.2.5% 97.5%.97.5%

OSA vs OBA 0.017173520 -0.001155010 0.03684901

OFA vs OBA 0.022813457 -0.001885303 0.05014984

OSA vs OFA -0.005639938 -0.031653321 0.01949180

check.names 0.000000000 0.000000000 0.00000000


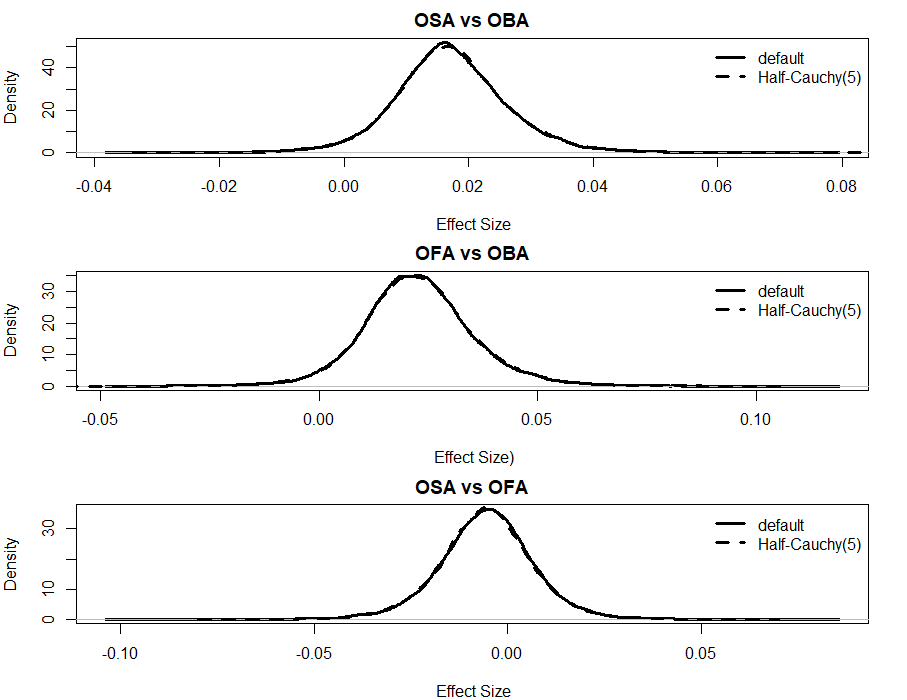


Default DIC:

Dbar pD DIC data points

19.31699 15.42317 34.74016 18.00000

Half-Cauchy DIC:

Dbar pD DIC data points

19.29664 15.43951 34.73616 18.00000
