# Supplementary material for: Opioid-sparing anesthesia versus opioid-free anesthesia for postoperative recovery quality in breast cancer surgery patients: A systematic review and Bayesian network meta-analysis
Source: PLoS One. 2025 Oct 24;20(10):e0334614. doi: 10.1371/journal.pone.0334614 (PMC12551851; doi:10.1371/journal.pone.0334614)
Supplement: S7 Text — Results of sensitivity tests. (DOCX) [file pone.0334614.s008.docx]

**Sensitivity analyses**

**Sensitivity analysis of effect size metrics：**

1. Empirical mean and standard deviation for each variable,

plus standard error of the mean:

Mean SD Naive SE Time-series SE

d.OBA.OFA 0.8858 0.5642 0.003990 0.003958

d.OBA.OSA 1.2857 0.2952 0.002088 0.002033

d.OSA.OFA -0.3999 0.5657 0.004000 0.003975

sd.d 1.0413 0.2344 0.001658 0.001694

2. Quantiles for each variable:

2.5% 25% 50% 75% 97.5%

d.OBA.OFA -0.2323 0.5258 0.8844 1.24430 2.0156

d.OBA.OSA 0.7003 1.0941 1.2810 1.47640 1.8689

d.OSA.OFA -1.5288 -0.7580 -0.4009 -0.03423 0.7109

sd.d 0.6805 0.8770 1.0055 1.16811 1.5897

-- Model fit (residual deviance):

Dbar pD DIC

17.53222 16.03672 33.56894

17 data points, ratio 1.031, I^2 = 9%

**Sensitivity analysis of QOR excluding studies with combined multiple arms：**

1. Empirical mean and standard deviation for each variable,

plus standard error of the mean:

Mean SD Naive SE Time-series SE

d.OBA.OFA 0.04996 0.009790 6.922e-05 6.996e-05

d.OBA.OSA 0.06004 0.005735 4.055e-05 4.086e-05

d.OSA.OFA -0.01008 0.009440 6.675e-05 6.739e-05

sd.d 0.01206 0.005607 3.964e-05 4.503e-05

2. Quantiles for each variable:

2.5% 25% 50% 75% 97.5%

d.OBA.OFA 0.029982 0.043966 0.05007 0.056229 0.069109

d.OBA.OSA 0.047651 0.056711 0.06033 0.063619 0.070925

d.OSA.OFA -0.028801 -0.015971 -0.01011 -0.004134 0.008496

sd.d 0.003412 0.008259 0.01122 0.014944 0.025302

-- Model fit (residual deviance):

Dbar pD DIC

25.44681 20.60782 46.05463

26 data points, ratio 0.9787, I^2 = 2%

**Sensitivity analysis of QOR excluding studies with combined multiple arms and high risk of bias：**
1. Empirical mean and standard deviation for each variable,

plus standard error of the mean:

Mean SD Naive SE Time-series SE

d.OBA.OFA 0.049331 0.010019 7.084e-05 7.251e-05

d.OBA.OSA 0.059055 0.006159 4.355e-05 4.389e-05

d.OSA.OFA -0.009724 0.009676 6.842e-05 6.895e-05

sd.d 0.012607 0.005984 4.231e-05 4.794e-05

2. Quantiles for each variable:

2.5% 25% 50% 75% 97.5%

d.OBA.OFA 0.028717 0.04312 0.049584 0.055716 0.068578

d.OBA.OSA 0.045609 0.05557 0.059387 0.063003 0.070259

d.OSA.OFA -0.028915 -0.01574 -0.009852 -0.003678 0.009634

sd.d 0.003542 0.00858 0.011660 0.015569 0.027132

-- Model fit (residual deviance):

Dbar pD DIC

23.55539 19.52281 43.07819

24 data points, ratio 0.9815, I^2 = 2%

**Sensitivity analysis of QOR excluding high-risk studies：**

1. Empirical mean and standard deviation for each variable,

plus standard error of the mean:

Mean SD Naive SE Time-series SE

d.OBA.OFA 0.043272 0.012173 8.608e-05 8.608e-05

d.OBA.OSA 0.048740 0.006364 4.500e-05 4.528e-05

d.OSA.OFA -0.005468 0.011997 8.483e-05 8.586e-05

sd.d 0.018539 0.005199 3.677e-05 3.677e-05

2. Quantiles for each variable:

2.5% 25% 50% 75% 97.5%

d.OBA.OFA 0.01928 0.03538 0.043160 0.051186 0.06742

d.OBA.OSA 0.03571 0.04470 0.048862 0.052864 0.06131

d.OSA.OFA -0.02910 -0.01315 -0.005598 0.002133 0.01836

sd.d 0.01076 0.01489 0.017778 0.021280 0.03104

-- Model fit (residual deviance):

Dbar pD DIC

30.62727 27.63514 58.26241

32 data points, ratio 0.9571, I^2 = 0%

**Sensitivity analysis of PONV excluding high-risk studies：**

1. Empirical mean and standard deviation for each variable,

plus standard error of the mean:

Mean SD Naive SE Time-series SE

d.OBA.OSA -1.2407 0.2994 0.002117 0.003462

d.OSA.OFA -1.8330 0.9282 0.006564 0.013154

d.OBA.OFA -3.0737 0.9238 0.006532 0.013358

sd.d 0.3072 0.2678 0.001894 0.003442

2. Quantiles for each variable:

2.5% 25% 50% 75% 97.5%

d.OBA.OSA -1.83971 -1.4317 -1.2340 -1.0435 -0.6651

d.OSA.OFA -3.89631 -2.3777 -1.7581 -1.2025 -0.1991

d.OBA.OFA -5.17072 -3.6169 -2.9919 -2.4402 -1.4938

sd.d 0.01007 0.1096 0.2412 0.4317 0.9789

-- Model fit (residual deviance):

Dbar pD DIC

14.81598 13.62821 28.44419

20 data points, ratio 0.7408, I^2 = 0%

**Sensitivity analysis of QOR excluding studies with combined multiple arms and high risk of bias：**

1. Empirical mean and standard deviation for each variable,

plus standard error of the mean:

Mean SD Naive SE Time-series SE

d.OBA.OSA -1.2374 0.3299 0.002333 0.003346

d.OSA.OFA -1.8206 0.9420 0.006661 0.012099

d.OBA.OFA -3.0580 0.9358 0.006617 0.011657

sd.d 0.3488 0.3015 0.002132 0.003651

2. Quantiles for each variable:

2.5% 25% 50% 75% 97.5%

d.OBA.OSA -1.90747 -1.4414 -1.2318 -1.0193 -0.5981

d.OSA.OFA -3.88637 -2.3985 -1.7676 -1.1844 -0.1229

d.OBA.OFA -5.12329 -3.6214 -2.9943 -2.4129 -1.3988

sd.d 0.01375 0.1263 0.2719 0.4845 1.1401

-- Model fit (residual deviance):

Dbar pD DIC

13.61163 12.51216 26.12379

18 data points, ratio 0.7562, I^2 = 0%

**Sensitivity analysis of QOR excluding studies with combined multiple arms：**

1. Empirical mean and standard deviation for each variable,

plus standard error of the mean:

Mean SD Naive SE Time-series SE

d.OBA.OSA -1.2917 0.2911 0.002059 0.003189

d.OSA.OFA -1.8062 0.9141 0.006464 0.012762

d.OBA.OFA -3.0979 0.9093 0.006430 0.012815

sd.d 0.3019 0.2554 0.001806 0.003100

2. Quantiles for each variable:

2.5% 25% 50% 75% 97.5%

d.OBA.OSA -1.88463 -1.4768 -1.2857 -1.099 -0.7276

d.OSA.OFA -3.82800 -2.3473 -1.7266 -1.181 -0.2136

d.OBA.OFA -5.09940 -3.6430 -3.0142 -2.477 -1.5264

sd.d 0.01144 0.1122 0.2404 0.421 0.9503

-- Model fit (residual deviance):

Dbar pD DIC

15.00439 13.62884 28.63323

20 data points, ratio 0.7502, I^2 = 0%
