# Supplementary material for: Opioid-sparing anesthesia versus opioid-free anesthesia for postoperative recovery quality in breast cancer surgery patients: A systematic review and Bayesian network meta-analysis
Source: PLoS One. 2025 Oct 24;20(10):e0334614. doi: 10.1371/journal.pone.0334614 (PMC12551851; doi:10.1371/journal.pone.0334614)
Supplement: S8 Text — Stability of findings via cumulative evidence. (DOCX) [file pone.0334614.s009.docx]

**Bayesian sequential meta-analysis**

To verify the stability of our findings, we conducted a Bayesian sequential meta-analysis incorporating 34 studies. The results demonstrate that the posterior mean stabilizes around 0.830 after initial fluctuations, with the 95% credible interval narrowing significantly from 0.196 to 0.004, indicating high precision. The posterior standard deviation decreases rapidly from 0.050 to 0.00097, reflecting a substantial reduction in uncertainty as evidence accumulates.

| Analysis Phase | Steps | Posterior Mean Range | Posterior SD Range | 95% CI Width | Key Characteristics |
| --- | --- | --- | --- | --- | --- |
| Initial Prior | 0 | 0.8480 | 0.05000 | 0.1960 | Weakly informative prior |
| Early Evidence | 1-5 | 0.8210–0.8940 | 0.00225–0.01266 | 0.0088–0.0496 | High volatility, rapid uncertainty reduction |
| Stabilization | 6-20 | 0.7902–0.8215 | 0.00110–0.00201 | 0.0043–0.0079 | Gradual convergence, moderate fluctuation |
| Final Convergence | 21-34 | 0.8219–0.8297 | 0.00097–0.00109 | 0.0038–0.0043 | Minimal change, evidence saturation |
| Final Result | 34 | 0.8297 | 0.00097 | 0.0038 | High precision estimate |


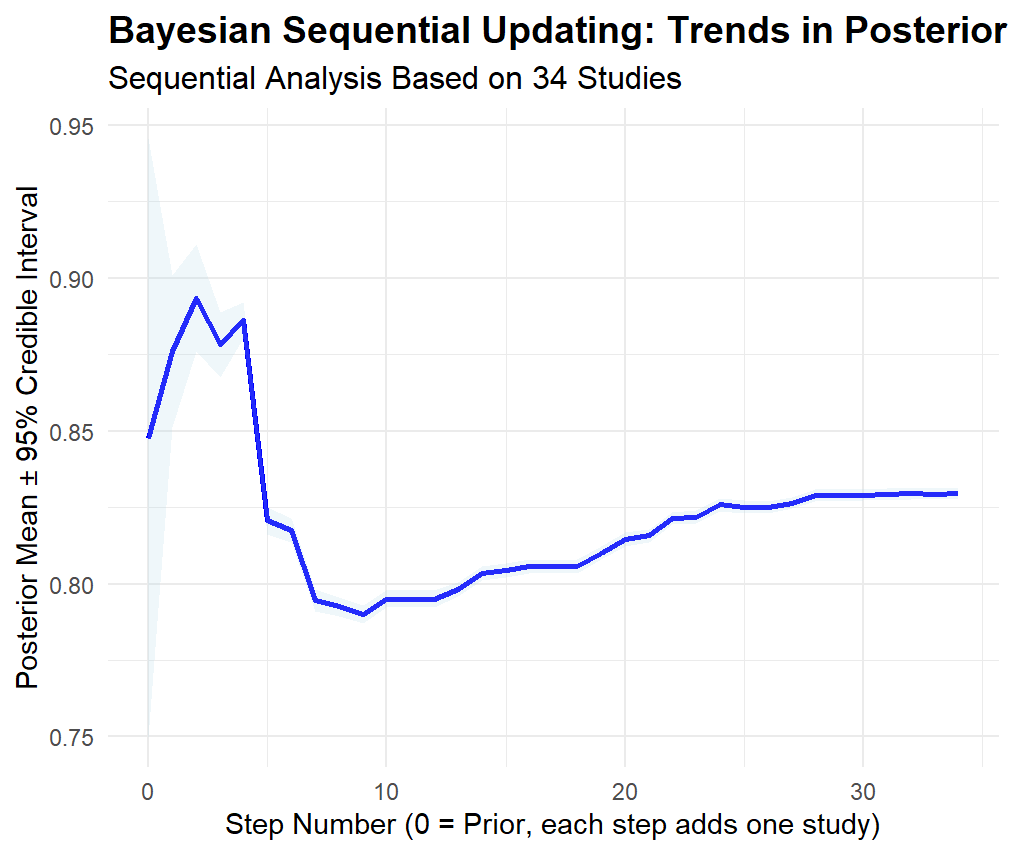


Figure 1: Illustrates the posterior mean trend, stabilizing at approximately 0.830, with the 95% credible interval shrinking markedly within the first few steps.


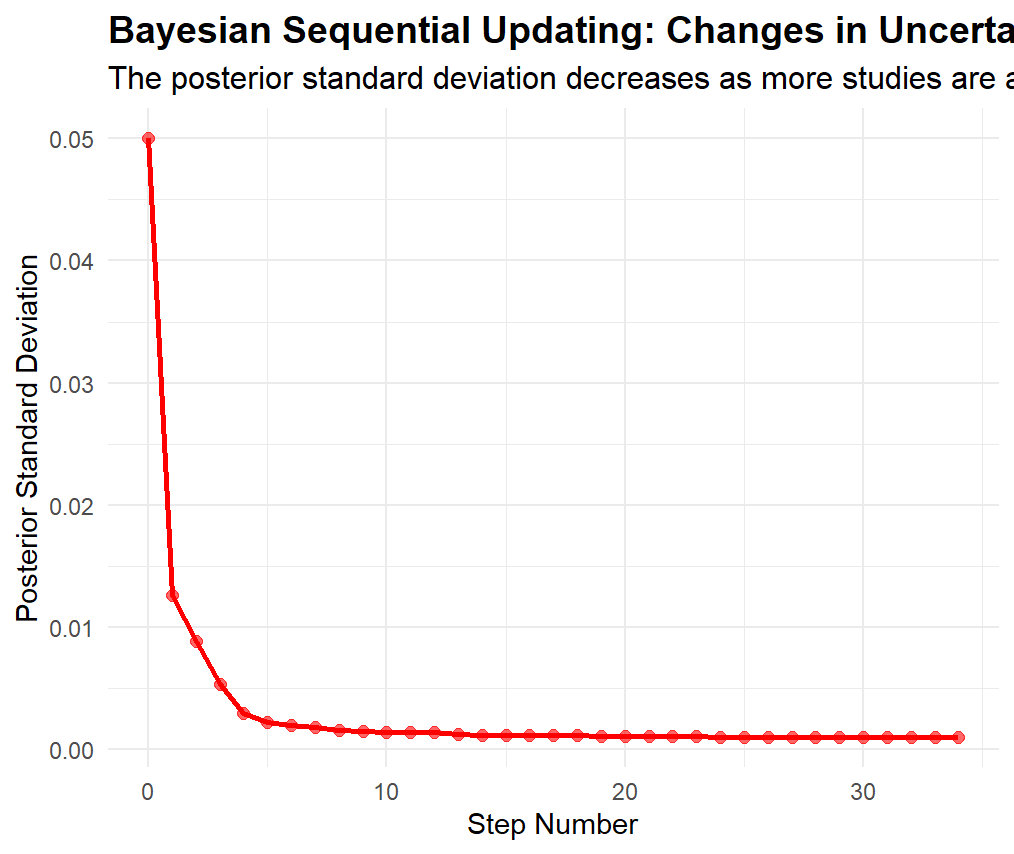


Figure 2: Shows the posterior standard deviation declining rapidly initially and leveling off after ~15 steps, confirming reduced uncertainty.


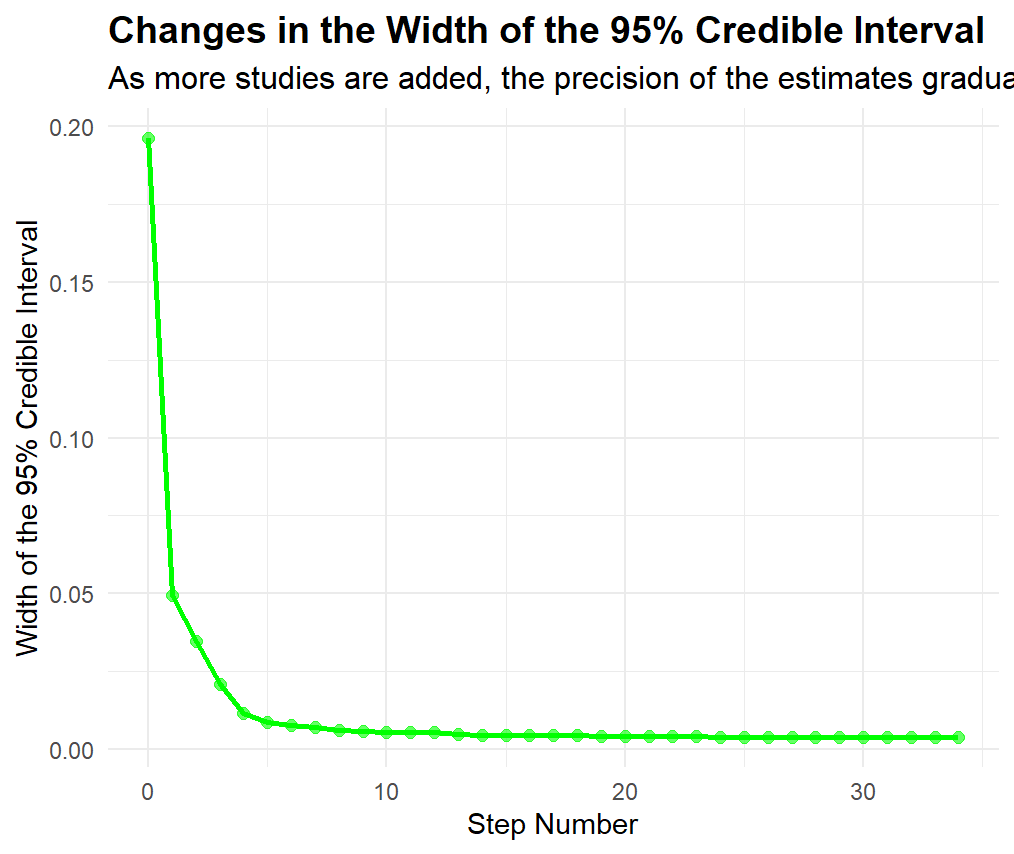


Figure 3: Displays the 95% credible interval width decreasing steadily, underscoring improved estimation precision over time.
